# CC002/Unc females are mouse models of exercise‐induced paradoxical fat response

**DOI:** 10.14814/phy2.13716

**Published:** 2018-06-19

**Authors:** Rachel C. McMullan, Martin T. Ferris, Timothy A. Bell, Vineet D. Menachery, Ralph S. Baric, Kunjie Hua, Daniel Pomp, Abbie E. Smith‐Ryan, Fernando Pardo‐Manuel de Villena

**Affiliations:** ^1^ Department of Genetics School of Medicine University of North Carolina at Chapel Hill Chapel Hill North Carolina; ^2^ Genetics and Molecular Biology Curriculum School of Medicine University of North Carolina at Chapel Hill Chapel Hill North Carolina; ^3^ Lineberger Comprehensive Cancer Center University of North Carolina at Chapel Hill Chapel Hill North Carolina; ^4^ Department of Epidemiology Gillings School of Global Public Health University of North Carolina at Chapel Hill Chapel Hill North Carolina; ^5^ Department of Exercise and Sport Science College of Arts and Sciences University of North Carolina at Chapel Hill Chapel Hill North Carolina

**Keywords:** Body composition, Collaborative Cross, genetic background, high intensity interval training, moderate intensity continuous training

## Abstract

Exercise results in beneficial health outcomes and protects against a variety of chronic diseases. However, U.S. exercise guidelines recommend identical exercise programs for everyone, despite individual variation in responses to these programs, including paradoxical fat gain. Experimental models of exercise‐induced paradoxical outcomes may enable the dissection of underlying physiological mechanisms as well as the evaluation of potential interventions. Whereas several studies have identified individual mice exhibiting paradoxical fat gain following exercise, no systematic effort has been conducted to identify and characterize models of paradoxical response. Strains from the Collaborative Cross (CC) genetic reference population were used due to its high levels of genetic variation, its reproducible nature, and the observation that the CC is a rich source of novel disease models, to assess the impact genetic background has on exercise responses. We identified the strain CC002/Unc as an exercise‐induced paradoxical fat response model in a controlled voluntary exercise study across multiple ages in female mice. We also found sex and genetic differences were consistent with this pattern in a study of forced exercise programs. These results provide a novel model for studies to determine the mechanisms behind paradoxical metabolic responses to exercise, and enable development of more rational personalized exercise recommendations based on factors such as age, sex, and genetic background.

## Introduction

Exercise is known to have numerous positive health benefits, plays a role in weight management and obesity prevention, and has the potential to reduce morbidity associated with chronic diseases (Egan and Zierath 2013; Drenowatz 2016). Although exercise reduces health risks, the effects on body composition are still unknown. Exercise is expected to reduce body mass and fat, but exercise‐induced weight loss is often less than expected (Drenowatz 2016). In both human and rodent populations, individual variation across a multitude of exercise‐induced responses occurs, with some individuals experiencing paradoxical responses (Goedecke et al. 2000; Swallow et al. 2001; Venables et al. 2005; Barwell et al. 2009; Garland et al. 2010; Karavirta et al. 2011; Bouchard et al. 2012; Mann et al. 2014). Even when exercise doses and energy expenditures are controlled, there is large individual variation in body mass and composition responses to exercise programs (Barwell et al. 2009; Kelly et al. 2011; Blundell et al. 2015). In fact, some humans gain weight (King et al. 2009; Drenowatz 2016) and gain body fat (Barwell et al. 2009) in response to exercise; and similar paradoxical responses have been observed in outbred mice (Kelly et al. 2011). This observed variation can be partially attributed to insufficient exercise dose, lack of adherence, and physiological and behavioral compensatory adaptations (e.g., energy intake, habitual activity levels, metabolic adaptations) (King et al. 2012; Drenowatz 2016). Furthermore, the individual variation in response to exercise treatments suggests genetic variation contributes to differences in exercise‐induced responses (Bouchard and Tremblay 1997; Phares et al. 2004; Barwell et al. 2009; Bray et al. 2009; Nehrenberg et al. 2009; Garland et al. 2010; Booth et al. 2012; Gordon et al. 2016). Initial studies in humans (Bouchard and Tremblay 1997; Phares et al. 2004; Bray et al. 2009; Garland et al. 2010; Mitchell et al. 2010) and rodents (Nehrenberg et al. 2009; Leamy et al. 2010; Kelly et al. 2011; Gordon et al. 2016) have suggested that genetics contribute to exercise‐induced body mass and composition responses.

U.S. guidelines for physical activity recommends the same exercise programs despite age or sex (U.S. Department of Health and Human Services, 2008). A moderate intensity continuous training (MICT) program consists of continuous exercise at a moderate intensity and closely resembles recommended physical activity guidelines. Alternatively, exercise programs can vary in intensity, such as high intensity interval training (HIIT), which consists of exercising at intervals of high intensity followed by short periods of low intensity or rest. HIIT programs are time‐efficient alternatives to MICT and have been shown to elicit rapid beneficial physiological responses (Helgerud et al. 2007; Gibala et al. 2012). Initial studies have demonstrated HIIT can efficiently and effectively reduce body fat (Boutcher 2011; Smith‐Ryan et al. 2016) and increase lean mass (Smith‐Ryan et al. 2015; Blue et al. 2018). However, individual variability in body composition responses with the presence of responders and nonresponders has been observed in HIIT, MICT and other exercise programs making it difficult to determine effective personalized exercise programs. Furthermore, initial evidence in humans suggests that physiological outcomes as a result of HIIT may be sex specific (Scalzo et al. 2014). It is unclear if or how genetics influences body composition in response to HIIT and MICT exercise programs (Tjonna et al. 2008; Nybo et al. 2010; Boutcher 2011; Gibala et al. 2012, 2014; Buchheit and Laursen 2013; Weston et al. 2014; Smith‐Ryan et al. 2015; Seldeen et al. 2018).

Given the complex interactions between physical activity, energy intake, body composition, as well as other variables, it is difficult to determine causal factors and successful exercise regimes that elicit beneficial responses to exercise in the human population. Sets of genetically distinct inbred mouse strains can be used to assess the impact of genetic responses for all of these traits while controlling environmental variables (Kostrzewa and Kas 2013; Kelly et al. 2015). Even though it is common in human studies, especially in women, to observe exercise‐induced body fat gain, no inbred mouse models exist which recapitulates these phenotypes. Most mouse studies (Nehrenberg et al. 2009; Leamy et al. 2010; Takeshita et al. 2012; Gordon et al. 2016) have only observed a standard response (body fat loss) to exercise, with the exception of some outbred mice presenting paradoxical responses (Kelly et al. 2011). A previous study used incipient Collaborative Cross (pre‐CC) mice to examine exercise‐related traits and observed ~17% of the pre‐CC lines had a paradoxical response to voluntary exercise (Mathes et al. 2011). Since biological replicates within each pre‐CC line were not tested, it remains unknown whether the observed paradoxical body composition responses were due to genetics, experimental noise or another underlying mechanism.

We utilized the CC population (Consortium CC, 2012; Threadgill and Churchill 2012; Bogue et al. 2015; Morgan and Welsh 2015; Srivastava et al. 2017) to determine if there are inbred strains with consistent paradoxical fat response to exercise. The CC was selected because of (1) the previously reported exercise‐induced paradoxical responses in the pre‐CC (Mathes et al. 2011); (2) its high genetic and phenotypic diversity (Ferris et al. 2013; Phillippi et al. 2013; Kelada et al. 2014; Rasmussen et al. 2014; Ferguson et al. 2015); (3) the possibility to generate biological replicates; and (4) it has proven to be a rich source for novel human disease models (Rasmussen et al. 2014; Rogala et al. 2014). We expected variation in exercise phenotypes among CC strains to be comparable to variation observed in the human population (Kelly et al. 2015), and to provide strain(s) that can serve as models of exercise‐induced paradoxical fat response.

Here, we report the results of three independent but related experiments in CC mice: a screen for variability in responses to voluntary exercise; validation of strain CC002/Unc as a model for paradoxical response; and finally, an evaluation of metabolic response for two different exercise programs. These studies aimed to identify potential CC strains with exercise‐induced paradoxical body composition response for model development and for understanding genetic background contribution on physiological responses to different exercise programs.

## Materials and Methods

Each section is divided into subsections for the following experiments: (1) Voluntary exercise screen; (2) CC002/Unc model validation; (3) Exercise program evaluation.

### Mice and exercise treatment

All mice were purchased from the Systems Genetics Core Facility (http://csbio.unc.edu/CCstatus/index.py) and housed in the Division of Comparative Medicine facilities at the University of North Carolina at Chapel Hill. All procedures performed within this experiment were approved by the University of North Carolina – Chapel Hill Institutional Animal Care and Use Committee (IACUC #15‐015). Mice were housed in a temperature‐controlled (23 ± 1°C) and humidity‐controlled vivarium with a standard 12:12 h light:dark cycle (lights on at 0700 h). Mice were allowed ad libitum access to standard laboratory chow (Tekland 2920X irradiated rodent chow, Envigo, Princeton, NJ; diet consists of 24% of calories from protein, 16% of calories from fat and 60% of calories from carbohydrates) and water.

#### Voluntary exercise screen

Female mice (*n* = 173 total mice; ~9 months ± 4 weeks) from 13 CC strains were used in this screen and are also part of an ongoing aging study at UNC. CC strains were selected based on availability of at least 15 age‐matched females (born April to October 2015). Females were selected due to the need to group house the mice during the aging process. Mice were assigned to experimental (voluntary exercise; *n* = 93) or control (no exercise; *n* = 80) treatment cohorts prior to the start of the experiment (*n* = 4–8 per strain and treatment). The experiment was performed in six batches spaced approximately 1 month apart each. During the experiment two mice died of unrelated causes, one CC040/TauUnc from the control cohort and one CC030/GeniUnc from the experimental cohort (these mice were only used in the baseline phenotypic analysis).

Mice in the experimental cohort were individually housed in standard laboratory cages with ad libitum access to attached running wheels (1.1 m circumference; Lafayette Industries Lafayette, IN; (McMullan et al. 2016)). Mice in the experimental cohort were given access to wheels immediately after transfer to cages with attached wheels. Wheel running data were recorded continuously in 1‐min interval over a 2‐week period using an automated activity wheel monitoring program (AWM, Lafayette Industries, Lafayette, IN). The following physical activity measurements were obtained for each day of wheel access: distance (total revolutions × 1.1 m), duration (cumulative 1‐min interval in which at least 1 revolution was recorded), and average speed (total distance/total duration; m/min) (Kelly et al. 2012). For days 11–12 of wheel access, the mean total distance, duration, and average speed were calculated for each mouse. Mice in the control cohort were group housed (or single housed in select cases when all other cage mates were assigned to the experimental cohort) for the 2 weeks.

#### CC002/Unc model validation

Female mice (*n* = 12 per strain; born September to October 2016) from CC002/Unc and CC037/TauUnc were used to assess robustness of the CC002/Unc model at a younger age (~4 months ± 2 weeks). Mice were assigned to control (CC002/Unc *n* = 3; CC037/TauUnc *n* = 5) or experimental (CC002/Unc *n* = 9; CC037/TauUnc *n* = 7) cohort. Both cohorts were acclimated for 2 weeks to single housing with attached wheels in same vivarium room but without access to wheels. After acclimation, mice in the experimental cohort were given ad libitum access to running wheels for 8 weeks. The control cohort did not have access to running wheels. Experimental procedures for running wheel data collection and physical activity calculations follow those detailed in the “Voluntary exercise screen” section above. Total weekly distance, total weekly duration and average weekly speed were calculated for each 2‐week interval of wheel access and were labeled 1, 2, 3, and 4 (weeks 1–2, weeks 3–4, weeks 5–6, weeks 7–8 of wheel access, respectively).

#### Exercise program evaluation

##### Strain selection

Four strains were selected based on the following criteria: (1) ability to run on treadmills, (2) fat response type to voluntary exercise, (3) endurance ability (MPD datasets: McMullan1, McMullan2, McMullan3; Experiment “Voluntary exercise screen”; all data was collected in older females). Selected strains were: CC002/Unc paradoxical fat responder, low endurance; CC027/GeniUnc paradoxical fat responder, high endurance; CC013/GeniUnc standard fat responder, low endurance; and CC037/TauUnc standard fat responder, high endurance.

##### Maximum endurance speed

To assess maximum endurance speed, mice from the selected strains (*n* = 3 per sex and strain) were group housed with the same sex and strain. Mice were acclimated to the treadmill (Exer 3/6, Columbus Instruments, Columbus, Ohio) over 3 days (Table S1). Then, mice were run to exhaustion using the following endurance protocol performed at 20° inclination: initial speed was 4 m/min, increased by 2 m/min every 2 min then at 12 m/min the speed increased by 1 m/min every minute. The endurance protocol was performed twice on each mouse on two separate days and maximum speed (m/min) was recorded for both days. Maximum speed was defined as the last speed the mouse was able to maintain steady treadmill running before failure. Failure was defined as the inability or refusal to run on the treadmill despite stimulus via shock grid or prodding. The mean maximum speed was calculated for each sex and strain combination (Table S2).

##### Exercise program protocol design

Strain‐ and sex‐specific training protocols (HIIT and MICT) were designed based on the measured mean maximum speeds. There were five separate exercise groups: (1) CC002/Unc females; (2) CC013/GeniUnc females; (3) CC027/GeniUnc and CC037/TauUnc females; (4) CC002/Unc and CC013/GeniUnc males; and (5) CC027/GeniUnc and CC037/TauUnc males. The HIIT protocols consisted of five intervals with 80% max speed for 4 min, 20% max speed for 1 min, and ten 30 sec transitions to decrease and increase speed between the different intensities. The MICT protocols were distance matched to the HIIT protocols and consisted of ~43 min duration at 50% max speed (Tables S2 and S3).

##### Exercise program evaluation

Mice (*n* = 252 total mice; *n* = minimum 8 per sex, strain and exercise treatment combination; age: 8–10 weeks at start; born between March to August 2016 and February to March 2017) from CC002/Unc, CC013/GeniUnc, CC027/GeniUnc and CC037/TauUnc were housed with the same strains and sex in groups of three. The experiment was performed in five batches. Every strain, sex, exercise program combination was represented at least once in each batch for batches 1–4. In order to increase biological replicates, an additional batch (batch 5) was added. Batch 5 consisted of CC002/Unc females, CC027/GeniUnc females, CC037/TauUnc females, CC027/GeniUnc males, and CC037/TauUnc males (for females HIIT *n* = 8 & MICT *n* = 8 per strain; for males HIIT *n* = 3 & MICT *n* = 3 per strain). Across all batches, within each home cage, there was one mouse randomly assigned to each of the three exercise programs (HIIT, MICT, and no exercise [NE]) to avoid confounding cage effects with exercise program effects (with the exception of batch 5 which consisted of only HIIT and MICT programs). In five cases (for batches 1–4) more than three mice were group housed in one cage.

Mice completed 5 weeks of exercise training on the Exer‐3/6 treadmills (Columbus Instruments, Columbus, Ohio). Mice assigned to both HIIT and MICT training protocols were acclimated to the treadmills for 3 days during the first week (Table S1). After acclimation, HIIT and MICT mice completed 4 weeks of training, three times a week of their respective training protocol (Table S3). All training occurred in the morning and mice were randomly assigned to a treadmill lane each training day. Compliance was tracked over the full 15 days of exercise training and mice with 50% or more noncompliant days were removed from statistical analysis. A mouse was considered noncompliant if it refused or was not able to continue regular treadmill running despite extra stimulus from shock and/or prodding. All noncompliant and dead mice were removed from the analysis and are not included in the presented data or sample sizes. Ten noncompliant mice were removed (two CC002/Unc females HIIT; two CC002/Unc males HIIT; three CC013/GeniUnc females HIIT; two CC027/GeniUnc females HIIT; one CC027/GeniUnc female MICT). Five mice died during the experiment and were removed (one CC013/GeniUnc female HIIT; one CC027/GeniUnc male MICT; one CC027/GeniUnc female HIIT; two CC037/TauUnc females HIIT).

### Metabolic measurements

In all experiments, body composition was assessed (during mornings 0700–1200 h) using whole‐body MRI (EchoMRI 3‐in‐1 Body Composition Analyzer, EchoMRI, Houston, TX) to determine fat and lean mass content (in grams) for each animal.

#### Voluntary exercise screen

Body mass and composition were measured immediately prior to the start of the experiment (prior to cage transfer), and following the 2‐week experiment for all cohorts. Food was weighed prior to and after the experiment for the experimental cohort. To prevent variation in food intake due to food wastage, any food spillage was collected and weighed (Koteja et al. 2003). Food intake for the control cohort was not tracked as a result of group housing.

#### CC002/Unc model validation

Body mass and composition were measured every 2 weeks over the 10 weeks of the experiment for all cohorts. Food was weighed at the same time points as body composition for both control and experimental cohorts.

#### Exercise program evaluation

Metabolic measurements and body mass and composition were measured prior to the start of the experiment and upon completion of exercise training. Metabolic measurements were assessed by indirect calorimetry (PhenoMaster, TSE systems, Chesterfield, MO). For each batch, mice were randomly assigned to a calorimetry batch (A, B, or C) and calorimetry cage (1–24). Calorimetry data recorded included: oxygen consumption (VO_2_; mL/h/kg), carbon dioxide output (VCO_2_; mL/h/kg), activity (counts), food weight (g), water volume (mL), and heat production (kcal/h/kg). Respiratory exchange ratio (RER; VCO_2_/VO_2_) was calculated at each collection point. To acclimate to single housing, mice were individually housed for 24 h prior to the start of the indirect calorimetry. Mice were then individually housed in calorimetry cages for 24 h and data were recorded every ~50 min for each calorimetry cage. After, mice were returned to their assigned group housing.

### Metabolic calculations

Body fat and lean mass percentages were calculated relative to body mass at each time point in every experiment. Body mass response was calculated as [(Post‐mass − pre‐mass)/pre‐mass] × 100. Body composition percentage response was calculated as [(postmeasurement % − premeasurement %)/premeasurement %] × 100. Body mass and composition responses for individual mice in the experimental cohort (or HIIT, MICT) were adjusted to the mean strain (or strain‐by‐sex) responses in the control cohort (or NE) to account for experimental variability between cohorts (e.g., adjusted body mass response = [individual body mass response−control cohort strain mean body mass response]; adjusted body fat % response = [individual body fat % response − control cohort strain mean body fat % response]). Negative values represent a loss and positive values represent a gain in response to treatment. Food intake was calculated as the differential between baseline and postexercise food weights (g). Adjusted food intake was calculated as the food intake relative to the baseline body mass.

#### Voluntary exercise screen

Body mass and composition responses for both cohorts were calculated for the 2 weeks of treatment. Adjusted body mass and composition responses were calculated for the experimental cohort.

#### CC002/Unc model validation

For each mouse, cumulative body mass and composition responses were calculated for every experimental time point interval (1–4; weeks 0–2, weeks 0–4, weeks 0–6, and weeks 0–8 of treatment, respectively). Adjusted cumulative body mass and composition responses were calculated for the experimental cohort at each time point interval. Adjusted food intake was calculated for each interval.

#### Exercise program evaluation

Body mass and composition responses were calculated for the 5 weeks of exercise program treatment. Adjusted body mass and composition responses were calculated for HIIT and MICT mice. Calorimetry data collected from 0700 to 1100 h (day) and 1900 to 2300 h (nocturnal) were used to calculate the following traits for each mouse for both day and nocturnal values (at baseline and posttreatment): mean VO_2_ intake, mean VCO_2_ output, mean RER, total activity, mean heat production, food intake, and water intake.

### Statistical analysis

All statistical analyses were performed in the R programming environment (https://cran.r-project.org). Descriptive statistics (mean, variance, coefficient of variance, standard deviation, and standard error) were calculated for phenotypes across CC strain (Tables S4–S8). Pearson's correlations were calculated for the relationship between body mass and composition responses and potential mediators (physical activity traits and adjusted food intake). Heritability of body mass and composition response in the voluntary exercise screen was measured by inter‐class correlation (icc) and the coefficient of genetic determination (cgd) (Petkova et al. 2008). In order to determine potential mediators of the physiological responses between strains, we utilized a nested ANOVA framework to identify the set of explanatory variables (e.g., treatment, genetic background, sex, metabolic responses), which robustly explain variation in the physiological responses.

### Data availability

All data are publically available at the Mouse Phenome Database (https://phenome.jax.org). Datasets McMullan1–3 contain forced endurance abilities, voluntary exercise abilities and body mass and composition responses to voluntary exercise in aged females from ~50 CC strains. These datasets were used to select strains for experiment 3 presented here. The data presented in this paper is available in datasets: McMullan4–7. Datasets McMullan4–7 contains all raw data and experimental variables for each individual mouse in each of the three separate experiments presented. CC strain information is located at: http://csbio.unc.edu/CCstatus/index.py (Morgan and Welsh 2015; Srivastava et al. 2017).

## Results

### Screen for voluntary exercise‐induced paradoxical body composition responders in aged CC females

In a screen of 13 CC strains, body mass and composition responses to 2 weeks of treatment (control or experimental) were measured in ~9‐month‐old females. Exercise had a significant effect on body mass and composition responses (*P* < 1.0 × 10^−5^), but genetic background had a greater contribution (Table 1). All responses were heritable (cgd = 31.0%, icc = 47.4% body mass response; cgd = 22.4%, icc = 36.8% body fat response; and cgd = 26.3%, icc = 41.7% lean mass response). Furthermore, there were significant genetic background‐by‐treatment interactions on body mass, body fat and lean mass responses (nominal *P* = 5.1 × 10^−11^, *P* = 3.5 × 10^−7^, and *P* = 1.9 × 10^−9^, respectively, Table 1).

**Table 1:** Nominal *P*‐values from nested ANOVA analysis of treatment cohort and genetic background effect on body mass and composition response. Base models included treatment cohort as a fixed effect. Additive models included treatment cohort and genetic background as an additive effect. Full models included treatment cohort effect, genetic background effect and their interaction

Response	Base versus additive model	Base versus full model	Additive versus full model
Body mass	1.35 × 10^−9^	5.14 × 10^−11^	8.18 × 10^−4^
Body fat%	1.19 × 10^−6^	3.53 × 10^−7^	0.011
Lean mass%	7.7 × 10^−8^	1.91 × 10^−9^	8.74 × 10^−4^

**Figure 1:**
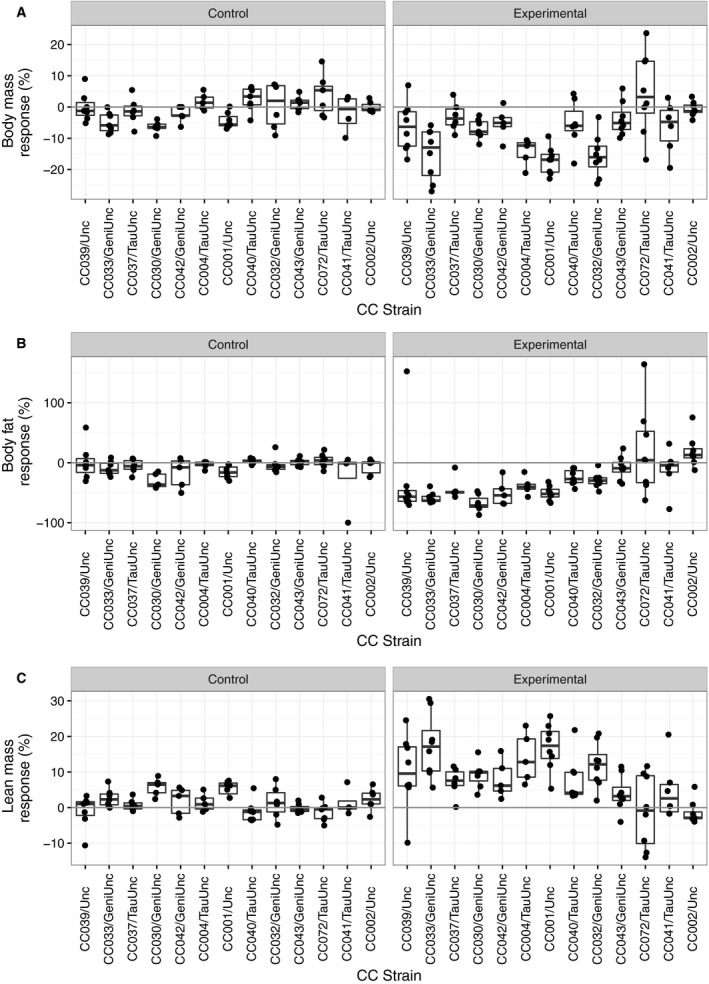
Body mass and composition response to 2 weeks of treatment in aged females across 13 CC strains. Body mass response (%) (A), body fat percentage response (%) (B) and lean mass percentage response (%) (C) over 2 weeks of treatment in both control and experimental treatment cohorts (*n* = 4–8 per treatment and strain; age ~9 months). Each dot represents an individual female mouse. Strains are ordered by median adjusted fat response.

**Figure 2:**
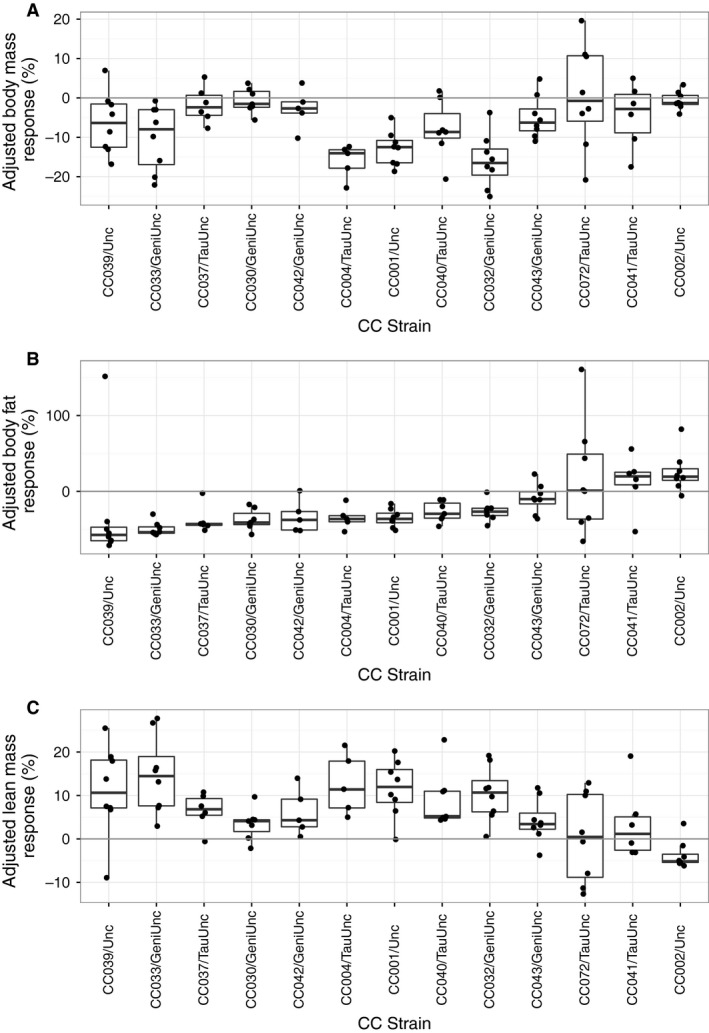
Exercise‐induced body mass and composition response in aged females across 13 CC strains. Adjusted body mass response (%) (A), adjusted body fat percentage response (%) (B) and adjusted lean mass percentage response (%) (C) to 2 weeks of wheel access in the experimental cohort (*n* = 5–8 per strain; age ~9 months). Responses are adjusted to strain mean responses in the control cohort. Each dot represents an individual female mouse. Strains are ordered by median adjusted fat response.

In the control cohort (no exercise), three strains had a significant standard body mass response (lost body mass), two strains had a significant standard body fat response (lost body fat), and three strains had a significant standard lean mass response (gained lean mass). In the experimental cohort (voluntary exercise), six CC strains had a significant body mass loss, seven strains had a significant standard body fat response, and nine CC strains had a significant standard lean mass response (Fig. 1, Table S4). In order to determine the effect of exercise independent of aging, the responses in the experimental cohort were adjusted to the mean strain responses in the control cohort. In the experimental cohort, six strains had a significant standard adjusted body mass response (Fig. 2A). Eight CC strains had a significant standard adjusted body fat response to exercise treatment. CC072/TauUnc had no significant change in mean body fat as there was great individual variability within the strain. CC002/Unc had a significant paradoxical adjusted fat response (mean: 25.6%; range: −5.78% to +82.06%, nominal *P* = 0.028) (Fig. 2B). Tukey's post hoc analysis revealed CC002/Unc experimental mice had unadjusted body fat responses significantly different from CC001/Unc, CC030/GeniUnc, CC033/GeniUnc, CC037/TauUnc, and CC042/GeniUnc experimental mice (adjusted *P* < 0.05). Eight CC strains had a significant standard adjusted lean mass response and one strain, CC002/Unc, had a significant paradoxical adjusted lean mass response (nominal *P* = 0.015) (Fig. 2C, Table S4).

Other traits varied significantly by genetic background (running distance nominal *P* = 0.0025; duration nominal *P* = 0.018; speed nominal *P* < 2.7 × 10^−6^; adjusted food intake nominal *P* = 0.0001) (Figs. S1 and S2, Table S5). Therefore, phenotypic correlations were used to assess whether these potential mediators were associated with body mass and composition responses. Running duration was significantly and negatively correlated with body mass (*r* = −0.211) and body fat response (*r* = −0.223). Mean speed was significantly correlated with body fat response (*r* = −0.206). All physical activity traits had significant positive correlations with lean mass response (distance *r* = 0.244, duration *r* = 0.266, speed *r* = 0.278). Adjusted food intake was significantly correlated with body mass response (*r* = 0.755), although this is not surprising since adjusted food intake is calculated by dividing food intake by baseline body mass. Adjusted food intake was also significantly correlated with lean mass response (*r* = −0.665) and body fat response (*r* = 0.411) (Table 2). While potential mediators were correlated with responses, genetic background had a more significant contribution than any mediator alone to body mass and composition responses. There was a significant genetic background‐by‐adjusted food intake interaction on fat response (nominal *P* = 0.018, Table 3).

**Table 2:** Pearson's correlations between body mass and composition response and potential mediators

Responses	Body mass	Body fat %	Lean mass %
Distance (days 11–12)	−0.144	−0.202	0.244
Duration (days 11–12)	−0.211	−0.223	0.266
Speed (days 11–12)	−0.17	−0.206	0.278
Adjusted food intake	0.755	0.411	−0.665
Food intake	0.584	0.514	−0.581

Significant correlations (nominal *P* < 0.05) are highlighted in gray.

**Table 3:** Nominal *P*‐values from nested ANOVA analysis of potential mediators and genetic background on body mass and composition response within the experimental cohort

Response	Base versus additive model	Base versus full model	Additive versus full model
Mediator: distance			
Body mass	2.33 × 10^−8^	3.70 × 10^−7^	0.095
Body fat%	7.16 × 10^−6^	0.0012	0.807
Lean mass%	4.325 × 10^−7^	3.56 × 10^−5^	0.42
Mediator: duration			
Body mass	5.35 × 10^−8^	3.78 × 10^−7^	0.06
Body fat%	8.27 × 10^−6^	0.0019	0.889
Lean mass%	1.52 × 10^−6^	4.66 × 10^−6^	0.056
Mediator: speed			
Body mass	4.697 × 10^−8^	9.53 × 10^−7^	0.126
Body fat%	2.633 × 10^−5^	0.0051	0.924
Lean mass%	5.426 × 10^−7^	1.39 × 10^−5^	0.219
Mediator: adjusted food intake			
Body mass	4.45 × 10^−8^	9.30 × 10^−6^	0.498
Body fat%	6.09 × 10^−7^	5.90 × 10^−7^	0.018
Lean mass%	1.606 × 10^−9^	5.19 × 10^−6^	0.905

Base models included a mediator as a fixed effect. Additive models included a mediator and genetic background as additive effects. Full models included a mediator effect, genetic background effect and their interaction.

### Further characterization of CC002/Unc as a model for exercise‐induced paradoxical fat response

To determine whether the CC002/Unc model of exercise‐induced paradoxical body composition response extended beyond aged females, the model was tested in younger females. The experiment was performed in ~4‐month‐old females from CC002/Unc and CC037/TauUnc, the latter strain had a standard response at ~9 months. There was no significant effect of treatment, genetic background or genetic background‐by‐treatment interaction on body mass or body composition response to 2 weeks of treatment in young CC002/Unc and CC037/TauUnc females. Despite the lack of statistical significance, the direction and magnitude of fat response to 2 weeks of exercise was consistent between young and old CC002/Unc females. Young CC002/Unc females in the experimental cohort had a 28.94% mean unadjusted gain of body fat (range: −14.7–70.7%) and a 19.73% mean adjusted gain of body fat (range: −23.9–61.5%). CC002/Unc mice had a lower baseline body fat percentage in young (mean 10.4%) compared to old (mean 16.8%) females. Additionally, adjusted food intake was greater in young females (mean 2.2) than old females (mean 1.85). Younger CC002/Unc mice ran approximately the same mean distance (4.51 km), but at lower mean speed (15.7 m/min) and greater mean duration (279.1 min) than old mice (mean distance 4.49 km, speed 17.8 m/min, duration 248.6 min) on days 11–12. Initial body fat response to 2 weeks of voluntary exercise in young CC037/TauUnc females was not the same direction or magnitude as body fat response observed in old females (young: 6.68% unadjusted, −1.93% adjusted; old: −43.64% unadjusted, −37.85% adjusted mean body fat response) (Fig. S3, Tables S4–S6).

**Figure 3:**
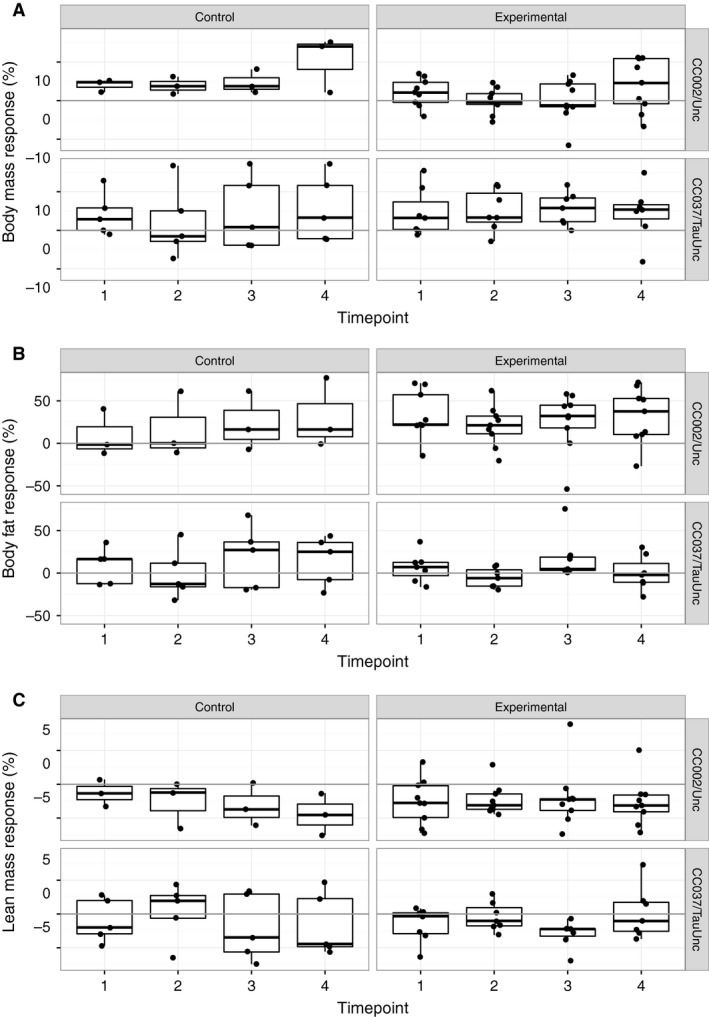
Cumulative body mass and composition response to treatment over 8 weeks in CC002/Unc and CC037/TauUnc. Body mass response (%) (A), body fat percentage response (%) (B) and lean mass percentage response (%) (C) for each 2 week interval of the experiment in both control and experimental treatment cohorts (*n* = 3–9 per treatment and strain; age ~4 months). Each dot represents an individual female mouse. Each response is calculated for a 2 week interval. Responses are represented as timepoint intervals: timepoint 0 (single housing acclimation), 1 (weeks 0–2 of treatment), 2 (weeks 2–4 of treatment), 3 (weeks 4–6 of treatment), and 4 (weeks 6–8 of treatment).

Cumulative body mass and composition response was also measured over 8 weeks of treatment to assess the effect of additional exercise on physiological responses. For cumulative body mass response, there was no significant effect of time point, treatment, genetic background and all their interactions. There was a significant additive effect of time point and genetic background on cumulative fat response (nominal *P* = 0.0018) and cumulative lean mass response (nominal *P* = 0.0471) demonstrating body composition responses varied by time point and genetic background. There was no significant effect of treatment on cumulative fat or lean mass response over time. Young experimental CC002/Unc females had an increase in paradoxical fat response over the 8 weeks of wheel access with a mean unadjusted cumulative fat response of 28.9, 20.3, 25.6, and 32.0% for weeks 0–2, 0–4, 0–6, and 0–8. In the CC002/Unc control cohort, mean unadjusted cumulative fat response increased over time intervals (9.2, 16.9, 23.6, and 30.9%). CC037/Tau mean cumulative fat response fluctuated over 8 weeks in experimental females (6.68, −5.6, 17.9, and 0.19% for weeks 0–2, 0–4, 0–6, and 0–8) and in control females (8.65, −0.77, 19.04, and 14.8%) (Fig. 3, Fig. S4, Table S6).

Total distance and duration in the CC002/Unc experimental females decreased during weeks 1–4 to weeks 4–8. Mean speed remained stable over 8 weeks of wheel access. CC037/TauUnc experimental females had stable running duration, but increased in mean speed and distance during the 8 weeks of exercise (Fig. S5, Table S7). Adjusted food intake increased during the first 2 weeks of wheel access in both cohorts and strains relative to food intake during single housing acclimation. Fluctuations in food intake were observed over the course of treatment in both cohorts and strains (Fig. S6). The fluctuations in physical activity levels and food intake over the course of treatment are important since energy expenditure and energy intake contribute to body mass and composition responses.

### Effect of exercise program on body composition response across both sexes and different genetic backgrounds

**Figure 4:**
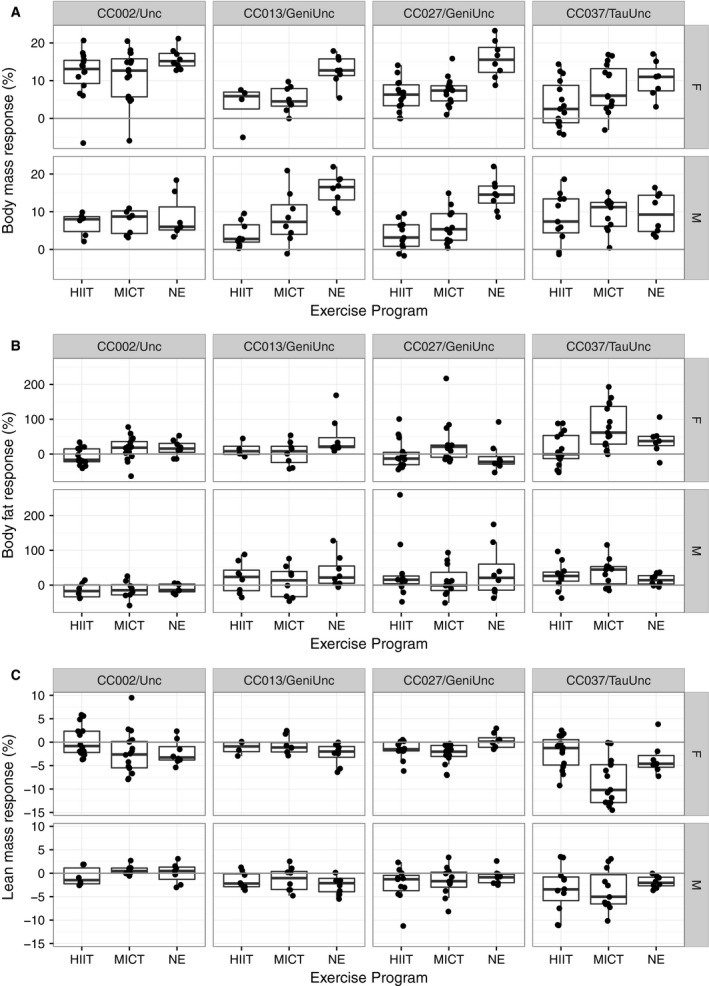
Body mass and composition response to exercise programs in four CC strains. Body mass response (%) (A), body fat percentage response (%) (B) and lean mass percentage response (%) (C) to 5 weeks of exercise program training (*n* = 4–16 per treatment, sex and strain; age ~8 weeks at start). Top panels are only female mice (F) and bottom panels are only male mice (M). Each dot represents an individual mouse.

**Figure 5:**
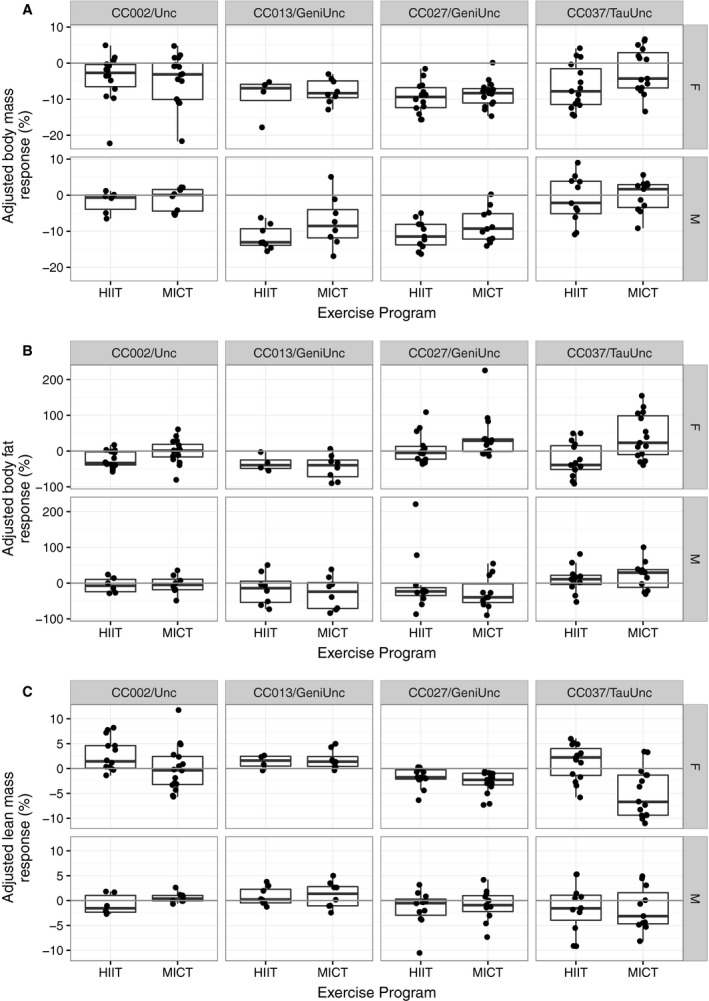
Adjusted body mass and composition response to exercise programs in four CC strains. Adjusted body mass response (%) (A), adjusted body fat percentage response (%) (B) and adjusted lean mass percentage response (%) (C) to 5 weeks of exercise program training (*n* = 4–16 per treatment, sex and strain; age ~8 weeks at start). Exercise programs include HIIT (high intensity interval training) and MICT (moderate intensity continuous training). Responses are adjusted to mean responses in matching strain and sex NE (no exercise) cohort. Top panels are only female mice (F) and bottom panels are only male mice (M). Each dot represents an individual mouse.

We selected four CC strains (see Materials and Methods) to measure the effects of genetic background, sex and two types of forced exercise programs (HIIT and MICT) on exercise‐induced metabolic responses. Overall, exercise programs, HIIT and MICT, significantly reduced body mass relative to NE programs (nominal *P* = 6.82 × 10^−12^; HIIT‐NE adjusted *P* < 1.0 × 10^−7^; MICT‐NE adjusted *P* = 3.0 × 10^−7^). Body mass response in mice exposed to HIIT was not significantly different from body mass response in mice exposed to MICT. Genetic background‐by‐exercise program had a significant interaction on body mass response (nominal *P* = 0.007) indicating that body mass response varied by exercise program dependent on genetic background. Body mass response was not significantly modified by sex; thus, both males and females had similar mass response (Figs. 4A and 5A, Tables 4 and 5, Table S8).

**Table 4:** Nominal *P* values from ANOVA of best‐fit linear models for body mass and composition responses

Response	Exercise program	Sex	Genetic background	Exercise program‐by‐sex interaction	Exercise program‐by‐genetic background interaction	Sex‐by‐genetic background interaction	Exercise program‐by‐sex‐by‐genetic background interaction
Body mass	1.20 × 10^−12^	NA	0.017	NA	0.007	NA	NA
Body fat	0.04226	0.699	6.738 × 10^−5^	0.025	0.048	0.034	0.577
Lean mass	0.0034	0.148	1.44 × 10^−^8	0.0037	0.0037	0.109	0.412

Body mass and composition responses are from mice exposed to either HIIT, MICT or NE (Exercise Program) and include both males and females (Sex) and four CC strains (Genetic Background). Best fit model are as follows: exercise program*genetic background (body mass response) or exercise program*sex*genetic background (body fat and lean mass response). Gray boxes represent significant nominal *P*‐values (*P* < 0.05). NA = not applicable.

**Table 5:** Adjusted *P*‐values from Tukey's post hoc analysis for best fit linear model for each body mass and composition response

Response	Exercise program			Sex	Genetic background					
	MICT versus HIIT	NE versus HIIT	NE versus MICT	M versus F	CC002 versus CC013	CC002 versus CC027	CC002 versus CC037	CC013 versus CC027	CC013 versus CC037	CC027 versus CC037
Body mass	0.057	<2.6 × 10^−16^	1.0 × 10^−7^	NA	0.077	0.054	0.026	0.999	0.998	0.991
Body fat	0.041	0.199	0.891	0.7	0.058	0.098	2.04 × 10^−5^	0.968	0.262	0.056
Lean mass	4.81 × 10^−3^	0.942	0.033	0.149	0.419	0.334	<2.6 × 10^−16^	0.999	2.50 × 10^−4^	3.25 × 10^−5^

Response	Exercise program‐by‐sex interaction								
	F			M			NE	HIIT	MICT
	MICT versus HIIT	NE versus HIIT	NE versus MICT	MICT versus HIIT	NE versus HIIT	NE versus MICT	M versus F	M versus F	M versus F
Body mass	NA	NA	NA	NA	NA	NA	NA	NA	NA
Body fat	4.96 × 10^−3^	0.3755	0.869	0.994	0.999	0.971	0.999	0.565	0.241
Lean mass	1.01 × 10^−^4	0.778	0.099	0.993	0.913	0.997	0.961	0.63	0.0175

Body mass and composition responses are from mice exposed to either HIIT, MICT or NE (Exercise Program) and include both males and females (Sex) and four CC strains (Genetic Background). Best fit model are as follows: exercise program*genetic background (body mass response) or exercise program*sex*genetic background (body fat and lean mass response). Gray boxes represent significant adjusted *P*‐values (*P* < 0.05). NA = not applicable.

**Table 6:** Analysis of metabolic traits and genetic background on body mass and composition response

Baseline metabolic trait	Body mass response	Body fat response	Lean mass response	Postmetabolic trait	Body mass response	Body fat response	Lean mass response
RER nocturnal	0.0048	0.0034	5.15 × 10^−5^	RER nocturnal	0.0176	0.0004	5.558 × 10^−10^
RER day	0.0068	0.0034	0.0001	RER day	0.0150	0.3552	0.0862
VO2 nocturnal	0.0017	0.0002	0.0001	VO2 nocturnal	9.357 × 10^−5^	0.0024	9.512 × 10^−5^
VO2 day	0.0171	0.0197	0.0026	VO2 day	0.0035	0.0092	8.007 × 10^−5^
VCO2 nocturnal	0.0005	0.0044	0.0010	VCO2 nocturnal	0.0057	0.0005	6.387 × 10^−7^
VCO2 day	0.0192	0.0121	9.226 × 10^−5^	VCO2 day	0.0144	0.1937	0.0054
Heat nocturnal	0.0013	0.0003	0.0017	Heat nocturnal	0.0003	0.0018	4.223 × 10^−5^
Heat day	0.0188	0.0208	0.0002	Heat day	0.0042	0.0194	0.0001
Activity nocturnal	0.0266	0.0680	0.0006	Activity nocturnal	0.0290	0.0200	0.0051
Activity day	0.0009	0.0170	6.118 × 10^−7^	Activity day	0.0074	0.0221	2.351 × 10^−5^
Food intake nocturnal	0.0394	0.0100	4.452 × 10^−5^	Food intake nocturnal	0.2746	0.0003	1.174 × 10^−7^
Food intake day	0.0007	0.0209	0.0041	Food intake day	0.0410	0.0639	0.0233
Water intake nocturnal	0.0072	0.1074	0.0007	Water intake nocturnal	0.0040	0.1490	0.0006
Water intake day	0.0006	0.0064	0.0014	Water intake day	0.0405	0.0622	0.0042

Nominal *P*‐values from partial *F* tests of a nested ANOVA analysis was performed comparing the best fit model with each metabolic variable subbed in for genetic background versus the best fit model including each metabolic variable. Best fit model are as follows: exercise program*genetic background (body mass response) or exercise program*sex*genetic background (body fat and lean mass response). Gray boxes represent nonsignificant *P*‐values (*P* > 0.05).

Exercise programs were suggestive of body fat percentage response (nominal *P* = 0.063), but the interaction between exercise program and sex had a significant effect on body fat percentage response (nominal *P* = 0.039). Specifically, body fat response varied between HIIT and MICT programs among females (adjusted *P* = 0.01). Furthermore, there was a significant genetic background‐by‐exercise program‐by‐sex interaction effect on body fat percentage response (nominal *P* = 0.0002). CC002/Unc females had a significant standard adjusted fat response to HIIT (mean −24.37%, nominal *P* = 0.002) but no significant change in adjusted fat response to MICT (mean −1.0%, nominal *P* = 0.906). CC027/GeniUnc females had a significant paradoxical adjusted fat response to MICT (mean 38.01%, nominal *P* = 0.028) but no significant adjusted fat response to HIIT (mean 6.55%, nominal *P* = 0.571). Unlike females, males demonstrated similar fat responses to both HIIT and MICT programs (Figs. 4B and 5B, Tables 4 and 5, Table S8).

Exercise program (nominal *P* = 0.011), and the interaction between exercise program and sex (nominal *P* = 0.0109), had a significant effect on lean mass percentage response. Again, the differences in lean mass response to HIIT and MICT programs in females were driving this significant interaction (adjusted *P* = 6.97 × 10^−4^). There was a significant genetic background‐by‐exercise program‐by‐sex interaction on lean mass percentage response (nominal *P* = 0.009). CC027/GeniUnc females had a significant paradoxical lean mass response to both HIIT (adjusted mean −1.70%, nominal *P* = 0.004) and MICT (adjusted mean −2.78%, nominal *P* = 0.0002) programs (Figs. 4C and 5C, Tables 4 and 5, Table S8).

All baseline metabolic variables (RER, VO_2_, VCO_2_, activity, heat, food intake, and water intake) during both nocturnal and daytime were under genetic control (nominal *P* < 0.05). Baseline metabolic variables were not more predictive than genetic background for body mass and composition responses to exercise programs. However, baseline nocturnal activity and nocturnal water intake were as predictive as genetic background for body fat response (Table 6). Pearson's correlations revealed baseline nocturnal activity was positively and significantly correlated with body fat response (*r* = 0.185, nominal *P* = 0.0035). These observations indicate baseline nocturnal activity levels are predictive of fat response to exercise. Whereas, baseline nocturnal water intake was not significantly correlated with body fat response (*r* = −0.067, nominal *P* = 0.29) indicating that water intake is not likely casual of body fat response to exercise and instead may be confounded. After exercise training, metabolic variables during both nocturnal and day time were under genetic control (nominal *P* < 0.05) with the exception of nocturnal RER (nominal *P* = 0.459), day VO2 (nominal *P* = 0.069) and day heat production (nominal *P* = 0.056). In some cases post metabolic traits were just as predictive as genetic background for body fat response (Table 6). This observation is likely due to the fact that the metabolic traits are genetically regulated.

## Discussion

### CC002/Unc is a model for exercise‐induced paradoxical body composition response

One previous study that reported an exercise‐induced paradoxical response in 17% of partially inbred mice (Mathes et al. 2011), only examined one individual mouse per genotype in the pre‐CC population, which limited the ability to assess genetic control of this trait. In contrast, our study used replicate inbred animals from the CC population with both sedentary control and voluntary exercise cohorts. The most significant finding was that CC002/Unc, one of the 13 CC strains screened in the initial study, had a voluntary exercise‐induced paradoxical body composition response among old females. It is possible that CC002/Unc overcompensates with food intake in response to exercise driving the observed paradoxical response, but this may not be the only factor or driving factor contributing to the paradoxical response. Food intake varied significantly by genetic background and there was a significant genetic background‐by‐adjusted food intake interaction on fat response. Thus, these findings suggest genetic background, including food intake, are driving the paradoxical response in old CC002/Unc females. For example, a standard response was observed in CC042/GeniUnc, which had similar levels of adjusted food intake and physical activity. Furthermore, a standard response occurred in CC039/Unc, even though CC039/Unc had similar adjusted food intake levels but lower physical activity levels than CC002/Unc. Stress from acclimation to single housing with wheel access could partially contribute to body mass and composition responses observed in the experimental cohort.

Young CC002/Unc females had consistent direction and magnitude of fat response to 2 weeks of voluntary exercise with old CC002/Unc females; however the treatment effect was not statistically significant at ~4 months due to low power. This is likely due to the variability present in the control and experimental treatment groups leading to overlap in the body composition response measurements between the groups in the younger mice. Interestingly, there was higher variance in body fat responses at ~4 months than at ~9 months. The higher levels of variability could be due to the ongoing alterations in body composition occurring at this age. Larger sample sizes should improve mean estimations and ultimately increase power. The younger CC002/Unc females had a lower baseline body fat percentage than the older mice (mean 10.4 and 16.8%, respectively). This is not surprising, as it is known that aging typically results in increased body fat, alterations in body composition and redistribution of fat (St‐Onge and Gallagher 2010; McMullan et al. 2016). The younger CC002/Unc females could have been undergoing alterations in body composition associated with aging over the 8 weeks of treatment. Other factors could have contributed to the higher level of variability in the young mice and lack of significant effect. Unlike the older control mice, the young control cohort was housed in the same vivarium and cages as the experimental cohort. The control cohort for the young CC002/Unc and CC037/TauUnc females could have been exposed to stress or other factors impacting body composition responses confounded in the experiment.

Young CC002/Unc females exposed to forced exercise programs (HIIT and MICT) did not have a paradoxical body composition response, as observed in the old and young CC002/Unc females exposed to voluntary exercise. Instead, CC002/Unc females had a significant standard adjusted fat response to HIIT and no significant change in adjusted fat response to MICT. It is important to note the forced endurance programs were performed three times a week over the course of 4 weeks and totaled ~480 m per session for CC002/Unc females; whereas, CC002/Unc females in the voluntary exercise treatment were running ~4.5 km on days 11–12 of wheel access. Thus, CC002/Unc females exposed to voluntary exercise had a greater frequency, duration and distance and varying intensity of exercise over 2 weeks than the females exposed to forced exercise programs. In rodents, both forced and voluntary exercise programs are used as a method to measure exercise abilities, exercise performance and other exercise‐related traits. Voluntary exercise is a self‐rewarding behavior and a complex trait that not only captures physical activity habits but also represents engagement in neural and physiological mechanisms required for the behavior (Kostrzewa and Kas 2013). While both forced and voluntary depend on common variables (e.g., physiological systems, organ function), there are distinct factors to each program including: psychological desire to run, fear, pain perception, shock avoidance, etc. (Lerman et al. 2002; Kelly and Pomp 2013; Kostrzewa and Kas 2013). Factors unique to voluntary exercise and their interaction with the CC002/Unc genetic background could be driving the observed exercise‐induced paradoxical body composition response. In conclusion, CC002/Unc females are a novel model mouse strain for voluntary exercise‐induced paradoxical body composition response. These findings strongly suggest that this response is due to unique physiological and metabolic conditions under genetic control. Future studies will be necessary to determine the underlying mechanism of paradoxical body composition responses and determine how other phenotypes respond to exercise in our identified models. Human studies have demonstrated a small subset of individuals have more than one adverse response phenotypes to exercise (Bouchard et al. 2012).

### Females have different body composition responses depending on exercise program and genetic background

Body composition responses to HIIT, MICT, and NE programs were examined in both males and females in four different genetic backgrounds. There was a significant genetic background‐by‐sex‐by‐exercise program interaction on both body fat and lean mass response to program. Specifically, females responded differently to HIIT and MICT programs, and the response further varied by genetic background. This finding indicates genetic background, sex, and exercise factors (e.g., intensity, duration) should be considered in design of exercise programs for humans. From this study, additional CC strains as potential models of paradoxical body composition responses to exercise were identified. In particular, both CC027/GeniUnc and CC037/TauUnc females had a significant paradoxical adjusted fat response to MICT programs. Additional studies utilizing these identified models of paradoxical responders and non‐responders will be necessary to identify underlying mechanistic pathways and genetic biomarkers predicting response to particular exercise programs. These studies will be important for informing the design of effective exercise programs for particular genetic populations and individuals.

In addition, the current study demonstrated that baseline metabolism, including RER, did not predict body mass and composition response. Genetic background instead predicted body mass and composition response in the four CC strains. RER is commonly used to indirectly determine the contribution of carbohydrates and lipids to energy expenditure. The contribution of these fuels can be affected by diet, muscle glycogen presence, exercise factors (intensity, duration) and training status (Venables et al. 2005; Ramos‐Jimenez et al. 2008). Individual variation in substrate oxidation during exercise has been observed in both trained and untrained individuals. In this study, baseline RER was not associated body composition response to exercise supporting prior findings in humans (Goedecke et al. 2000).

### Within strain individual variability was observed across phenotypes

Even though these studies were performed in mouse strains that were almost fully inbred (Srivastava et al. 2017), variance in all phenotypes and variability in levels of variance across CC strains was observed (Tables S4–S8). This is not surprising since individual variability in exercise‐related phenotypes in inbred strains has previously been observed (Lerman et al. 2002; Mathes et al. 2011). In particular, large individual variability in body fat percentage response, physical activity traits, and adjusted food intake occurred within CC072/TauUnc. It is possible that the differences in adjusted food intake, in combination with the differences in physical activity levels were driving the observed differences in fat response. In addition, CC072/TauUnc may be more susceptible to environmental influences (e.g., life history) or epigenetic influence (e.g., in utero environment) that were unaccounted for in the study design. The large individual variability observed in CC072/TauUnc was unlikely caused by segregating regions of the strain's genome since only 0.8% of the genome was segregating in this strain 2 years ago (Srivastava et al. 2017).

### Concluding remarks

Despite significant health burdens and public interest in understanding and optimizing exercise regimes within the human population, relatively little is known about the genetic architecture and control of the diverse behavioral, metabolic and physiological responses that converge to drive successful response to exercise. This study used mouse strains from the CC population to identify and develop mouse model(s) of exercise‐induced paradoxical fat responders. Genetic variation in the CC resulted in phenotypic diversity in exercise‐related traits. The presence of outliers in body composition response to exercise in a small subset of CC strains, further supports that the CC population is a rich source for new models of human traits (Rogala et al. 2014; Kelly et al. 2015).

CC002/Unc was identified as a model for paradoxical body composition response under certain conditions (females, voluntary exercise, significance of effect varies by age). Voluntary exercise‐induced body mass and composition responses were driven by genetic background independent of physical activity levels further supporting the importance of genetic background on exercise‐induced responses (Nehrenberg et al. 2009). Lastly, this study demonstrated a significant genetic background‐by‐sex‐by‐exercise program interaction on body composition response. Specifically, HIIT elicited more beneficial body composition responses than MICT programs in females dependent on genetic background. It will be vital to consider genetic background, sex and age in the design of effective exercise programs in the human populations.

## Data Accessibility

## Supporting information




**Table S1.** Treadmill protocols for 3 days of acclimation.
**Table S2.** Maximum endurance speed for each CC strain and sex.
**Table S3.** Exercise program treadmill protocols for HIIT and MICT for each of the 5 exercise groups.
**Table S4.** Descriptive statistics for body mass and composition responses across 13 CC strains.
**Table S5.** Descriptive statistics for physical activity traits in the experimental cohort across 13 CC strains.
**Table S6.** Descriptive statistics for cumulative body mass and composition response over 8 weeks of treatment.
**Table S7.** Descriptive statistics of physical activity traits in CC002/Unc and CC037/TauUnc young females.
**Table S8.** Descriptive statistics for body mass and composition response across four CC strains, both sexes and three exercise training programs.

**Figure S1.** Physical activity traits in aged females across 13 CC strains.
**Figure S2.** Adjusted food intake in experimental cohort across 13 CC strains.
**Figure S3.** Adjusted body mass and composition response to 2 weeks of exercise in young and old CC002/Unc and CC037/TauUnc female mice.
**Figure S4.** Cumulative adjusted exercise‐induced body mass and composition response over 8 weeks in CC002/Unc and CC037/TauUnc female mice.
**Figure S5.** Physical activity traits over 8 weeks of wheel access in CC002/Unc and CC037/TauUnc female mice.
**Figure S6.** Adjusted food intake over 10 weeks of treatment in CC002/Unc and CC037/TauUnc female mice.
