# Supplementary material for: CC002/Unc females are mouse models of exercise‐induced paradoxical fat response
Source: Physiol Rep. 2018 Jun 19;6(12):e13716. doi: 10.14814/phy2.13716 (PMC6009762; doi:10.14814/phy2.13716)
Supplement: Supplementary file 1 — Table S1. Treadmill protocols for 3 days of acclimation. Table S2. Maximum endurance speed for each CC strain and sex. Table S3. Exercise program treadmill protocols for HIIT and MICT for each of the 5 exercise groups. Table S4. Descriptive statistics for body mass and composition responses across 13 CC strains. Table S5. Descriptive statistics for physical activity traits in the experimental cohort across 13 CC strains. Table S6. Descriptive statistics for cumulative body mass and composition response over 8 weeks of treatment. Table S7. Descriptive statistics of physical activity traits in CC002/Unc and CC037/TauUnc young females. Table S8. Descriptive statistics for body mass and composition response across four CC strains, both sexes and three exercise training programs. [file PHY2-6-e13716-s001.pdf]

Supporting Table 1: Treadmill protocols for three days of acclimation. Interval length represents the length of each interval in seconds. Start speed is the speed (m/min) at which the interval begins at and end speed is the speed at which the interval ends. Interval distance is the distance (m) covered during each interval. Day 1 covers 129 m total, day 2 covers 185m total and day 3 covers 290.5m.

			Acclimation Day 1			
Angle of Inclination	5^0					
**Step**	**Start Speed (m/min)**	**End Speed (m/min)**	**Interval Length (s)**	**Interval Distance (m)**	**Total distance accumulated after this step (m)**	**Total Running Time (at end of this step; min)**
1	0	3	30	0.75	0.75	0.5
2	3	6	90	6.75	7.5	2
3	6	9	180	22.5	30	5
4	9	9	600	90	120	15
5	9	0	120	9	129	17

			Acclimation Day 2			
Angle of Inclination	10^0					
**Step**	**Start Speed (m/min)**	**End Speed (m/min)**	**Interval Length (s)**	**Interval Distance (m)**	**Total distance accumulated after this step (m)**	**Total Running Time (at end of this step; min)**
1	0	3	30	0.75	0.75	0.5
2	3	6	90	6.75	7.5	2
3	6	9	180	22.5	30	5
4	9	9	600	90	120	15
5	9	16	120	25	145	17
6	16	16	120	32	177	19
7	16	0	60	8	185	20

			Acclimation Day 3			
Angle of Inclination	15^0					
**Step**	**Start Speed (m/min)**	**End Speed (m/min)**	**Interval Length (s)**	**Interval Distance (m)**	**Total distance accumulated after this step (m)**	**Total Running Time (at end of this step; min)**
1	0	3	30	0.75	0.75	0.5
2	3	6	90	6.75	7.5	2
3	6	9	180	22.5	30	5
4	9	9	60	9	39	6
5	9	16	60	12.5	51.5	7
6	16	16	180	48	99.5	10
7	16	18	180	51	150.5	13
8	18	18	120	36	186.5	15
9	18	20	180	57	243.5	18
10	20	20	120	38	281.5	20
11	18	0	60	9	290.5	21

Supporting Table 2: Maximum endurance speed for each CC strain and sex. Max Speed (m/min) for each mouse is reported for both testing days. Mean strain speed (m/min) was calculated as a mean for each strain and sex across all maximum endurance speeds collected within the strain-by-sex groups. Protocol group represents the strains and sexes that will be running through exercise programs together and are matched by sex and maximum endurance speed abilities. Protocol group mean speed (m/min) is calculated as the mean speed for protocol groups with more than one strain. Speeds for the training programs are calculated using the protocol group mean speed.

CC Strain	Sex	Endurance Testing Day	Max Speed (m/min)			Mean Strain Speed (m/min)	Protocol Group	Protocol Group Mean Speed (m/min)	Calculated Speeds for Training Protocols		
			Mouse 1	Mouse 2	Mouse 3				80%	20%	50%
CC002/Unc	F	1	23	25	26	24.17	1	24.17	19.33	4.83	12.08
		2	25	23	23						
CC013/GeniUnc	F	1	39	28	30	33.67	2	33.67	26.93	6.73	16.83
		2	39	38	28						
CC027/GeniUnc	F	1	41	40	39	40.50	3	38.42	30.73	7.68	19.21
		2	43	38	42						
CC037/TauUnc	F	1	38	32	33	36.33	3				
		2	39	38	38						
CC002/Unc	M	1	26	21	25	24.00	4	25.58	20.47	5.12	12.79
		2	27	20	25						
CC013/GeniUnc	M	1	27	29	29	27.17	4				
		2	25	27	26						
CC027/GeniUnc	M	1	28	27	27	27.33	5	28.08	22.47	5.62	14.04
		2	30	23	29						
CC037/TauUnc	M	1	32	30	26	28.83	5				
		2	31	29	25						

Supporting Table 3: Exercise Program Treadmill Protocols for HIIT and MICT for each of the 5 exercise groups. Interval length represents the length of each interval in seconds. Start speed is the speed (m/min) at which the interval begins at and end speed is the speed at which the interval ends. Interval distance is the distance (m) covered during each interval. For each exercise group the total time (minutes) and distance (m) is listed for both HIIT and MICT programs. All HIIT programs are 33 minutes long and all MICT programs are 42 minutes long. Total distance (for both HIIT and MICT programs) for each exercise group are as follows: CC002/Unc females 480m; CC013/GeniUnc females 670m; CC027/GeniUnc and CC037/TauUnc females 764m; CC002/Unc and CC013/GeniUnc males 509m; CC027/GeniUnc and CC037/TauUnc males 559m.

HIIT Protocols

	**Protocol Group**	**1**			**2**			**3**			**4**			**5**		
	**Interval Length (s)**	**Start Speed (m/min)**	**End Speed (m/min)**	**Interval Distance**	**Start Speed (m/min)**	**End Speed (m/min)**	**Interval Distance**	**Start Speed (m/min)**	**End Speed (m/min)**	**Interval Distance**	**Start Speed (m/min)**	**End Speed (m/min)**	**Interval Distance**	**Start Speed (m/min)**	**End Speed (m/min)**	**Interval Distance**
	60	0.00	4.83	2.42	0.00	6.73	3.37	0.00	7.68	3.84	0.00	5.12	2.56	0.00	5.62	2.81
	60	4.83	4.83	4.83	6.73	6.73	6.73	7.68	7.68	7.68	5.12	5.12	5.12	5.62	5.62	5.62
	30	4.83	19.30	6.03	6.73	26.93	8.42	7.68	30.73	9.60	5.12	20.47	6.40	5.62	22.47	7.02
Intervals 1 - 5	240	19.30	19.30	77.20	26.93	26.93	107.72	30.73	30.73	122.92	20.47	20.47	81.88	22.47	22.47	89.88
	30	19.30	4.83	6.03	26.93	6.73	8.42	30.73	7.68	9.60	20.47	5.12	6.40	22.47	5.62	7.02
	60	4.83	4.83	4.83	6.73	6.73	6.73	7.68	7.68	7.68	5.12	5.12	5.12	5.62	5.62	5.62
	60	4.83	0.00	2.42	6.73	0.00	3.37	7.68	0.00	3.84	5.12	0.00	2.56	5.62	0.00	2.81

MICT Protocols

	**Protocol Group**	**1**			**2**			**3**			**4**			**5**		
	**Interval Length (s)**	**Start Speed (m/min)**	**End Speed (m/min)**	**Interval Distance**	**Start Speed (m/min)**	**End Speed (m/min)**	**Interval Distance**	**Start Speed (m/min)**	**End Speed (m/min)**	**Interval Distance**	**Start Speed (m/min)**	**End Speed (m/min)**	**Interval Distance**	**Start Speed (m/min)**	**End Speed (m/min)**	**Interval Distance**
	120	0.00	12.08	12.08	0.00	16.83	16.83	0.00	19.21	19.21	0.00	12.79	12.79	0.00	14.04	14.04
Interval 1	2295	12.08	12.08	462.06	16.83	16.83	643.75	19.21	19.21	734.78	12.79	12.79	489.22	14.04	14.04	537.03
	60	12.08	0.00	6.04	16.83	0.00	8.42	19.21	0.00	9.61	12.79	0.00	6.40	14.04	0.00	7.02

Supporting Table 4. Descriptive statistics for body mass and composition responses across 13 CC strains.

			Body Mass Response					Body Fat % Response					Lean Mass % Response				
CCStrain	Treatment	n	Mean	Variance	Coefficient of Variance	Std Deviation	Std Error	Mean	Variance	Coefficient of Variance	Std Deviation	Std Error	Mean	Variance	Coefficient of Variance	Std Deviation	Std Error
CC001/Unc	Control	7	-4.33	6.82	-1.57	2.61	0.99	-15.64	111.94	-7.16	10.58	4.00	5.41	4.18	0.77	2.04	0.77
CC002/Unc	Control	6	-0.03	3.18	-117.97	1.78	0.73	-6.48	168.53	-26.03	12.98	5.30	2.30	9.91	4.31	3.15	1.29
CC004/TauUnc	Control	5	1.72	7.17	4.16	2.68	1.20	-3.82	34.20	-8.94	5.85	2.62	1.42	6.15	4.34	2.48	1.11
CC030/GeniUnc	Control	5	-6.37	3.71	-0.58	1.93	0.86	-30.30	157.02	-5.18	12.53	5.60	5.80	6.40	1.10	2.53	1.13
CC032/GeniUnc	Control	6	0.47	54.06	115.57	7.35	3.00	-2.96	221.58	-74.82	14.89	6.08	1.49	21.02	14.13	4.58	1.87
CC033/GeniUnc	Control	7	-4.97	10.99	-2.21	3.31	1.25	-9.23	138.09	-14.96	11.75	4.44	2.69	6.37	2.37	2.52	0.95
CC037/TauUnc	Control	6	-1.28	19.23	-15.06	4.39	1.79	-5.80	138.22	-23.85	11.76	4.80	0.75	2.89	3.86	1.70	0.69
CC039/Unc	Control	7	0.04	22.43	577.93	4.74	1.79	0.80	866.84	1080.80	29.44	11.13	-1.06	22.16	-20.90	4.71	1.78
CC040/TauUnc	Control	8	2.64	14.73	5.59	3.84	1.36	2.37	16.53	6.98	4.07	1.44	-1.06	10.19	-9.65	3.19	1.13
CC041/TauUnc	Control	4	-1.96	37.10	-18.95	6.09	3.05	-24.17	2563.64	-106.06	50.63	25.32	1.43	15.28	10.70	3.91	1.95
CC042/GeniUnc	Control	5	-2.40	6.83	-2.85	2.61	1.17	-16.82	644.75	-38.32	25.39	11.36	1.86	14.80	7.93	3.85	1.72
CC043/GeniUnc	Control	7	1.15	4.80	4.19	2.19	0.83	0.97	39.91	41.14	6.32	2.39	-0.21	1.59	-7.44	1.26	0.48
CC072/TauUnc	Control	7	3.97	40.49	10.21	6.36	2.40	3.35	136.61	40.84	11.69	4.42	-1.28	6.76	-5.29	2.60	0.98
CC001/Unc	Experimental	8	-17.15	19.63	-1.14	4.43	1.57	-50.75	141.58	-2.79	11.90	4.21	16.99	42.78	2.52	6.54	2.31
CC002/Unc	Experimental	8	-0.70	5.17	-7.43	2.27	0.80	19.17	690.32	36.02	26.27	9.29	-1.40	10.64	-7.63	3.26	1.15
CC004/TauUnc	Experimental	5	-14.32	18.76	-1.31	4.33	1.94	-38.49	227.00	-5.90	15.07	6.74	14.01	49.17	3.51	7.01	3.14
CC030/GeniUnc	Experimental	8	-7.07	10.36	-1.47	3.22	1.14	-67.54	192.33	-2.85	13.87	4.90	9.20	13.93	1.51	3.73	1.32
CC032/GeniUnc	Experimental	8	-15.55	47.25	-3.04	6.87	2.43	-29.13	159.90	-5.49	12.65	4.47	11.88	40.13	3.38	6.33	2.24
CC033/GeniUnc	Experimental	8	-15.07	68.62	-4.55	8.28	2.93	-58.86	82.49	-1.40	9.08	3.21	17.39	80.84	4.65	8.99	3.18
CC037/TauUnc	Experimental	6	-3.04	21.08	-6.92	4.59	1.87	-43.64	313.47	-7.18	17.71	7.23	7.25	16.40	2.26	4.05	1.65
CC039/Unc	Experimental	8	-6.31	61.27	-9.71	7.83	2.77	-31.03	5594.76	-180.28	74.80	26.45	10.03	109.66	10.93	10.47	3.70
CC040/TauUnc	Experimental	7	-5.37	55.68	-10.37	7.46	2.82	-24.53	177.63	-7.24	13.33	5.04	8.11	45.09	5.56	6.72	2.54
CC041/TauUnc	Experimental	6	-6.43	68.22	-10.60	8.26	3.37	-11.77	1307.25	-111.10	36.16	14.76	4.88	71.00	14.54	8.43	3.44
CC042/GeniUnc	Experimental	5	-5.21	25.76	-4.95	5.08	2.27	-49.98	470.84	-9.42	21.70	9.70	8.04	29.33	3.65	5.42	2.42
CC043/GeniUnc	Experimental	8	-3.81	28.77	-7.54	5.36	1.90	-8.23	369.16	-44.85	19.21	6.79	3.99	24.99	6.26	5.00	1.77
CC072/TauUnc	Experimental	8	4.35	173.68	39.94	13.18	4.66	19.69	5309.06	269.65	72.86	25.76	-0.95	105.86	-111.75	10.29	3.64

Supporting Table 5. Descriptive statistics for physical activity traits in the experimental cohort across 13 CC strains.

			Mean Distance Days 11-12					Mean Duration Days 11-12					Mean Speed Days 11-12				
CCStrain	Treatment	n	Mean	Variance	Coefficient of Variance	Std Deviation	Std Error	Mean	Variance	Coefficient of Variance	Std Deviation	Std Error	Mean	Variance	Coefficient of Variance	Std Deviation	Std Error
CC001/Unc	Experimental	8	3.84	3.95	1.03	1.99	0.70	258.75	11,878.50	45.91	108.99	38.53	14.20	3.60	0.25	1.90	0.67
CC002/Unc	Experimental	8	4.49	2.06	0.46	1.44	0.51	248.63	4,647.84	18.69	68.18	24.10	17.79	8.07	0.45	2.84	1.00
CC004/TauUnc	Experimental	5	7.00	4.89	0.70	2.21	0.99	345.30	6,914.95	20.03	83.16	37.19	19.81	8.53	0.43	2.92	1.31
CC030/GeniUnc	Experimental	8	2.78	1.52	0.55	1.23	0.44	181.36	4,121.14	22.72	64.20	22.70	14.86	7.43	0.50	2.73	0.96
CC032/GeniUnc	Experimental	8	3.47	3.93	1.13	1.98	0.70	348.06	31,489.03	90.47	177.45	62.74	9.16	6.53	0.71	2.56	0.90
CC033/GeniUnc	Experimental	8	5.20	4.80	0.92	2.19	0.77	280.50	16,884.64	60.19	129.94	45.94	18.68	10.47	0.56	3.24	1.14
CC037/TauUnc	Experimental	6	6.65	9.81	1.47	3.13	1.28	362.33	15,623.57	43.12	124.99	51.03	17.31	19.60	1.13	4.43	1.81
CC039/Unc	Experimental	8	2.83	0.92	0.32	0.96	0.34	261.81	3,294.92	12.59	57.40	20.29	11.01	12.93	1.17	3.60	1.27
CC040/TauUnc	Experimental	7	6.49	8.85	1.36	2.98	1.12	345.36	9,620.48	27.86	98.08	37.07	18.80	32.03	1.70	5.66	2.14
CC041/TauUnc	Experimental	6	1.05	3.83	3.65	1.96	0.80	89.08	11,416.94	128.16	106.85	43.62	5.90	31.62	5.36	5.62	2.30
CC042/GeniUnc	Experimental	5	4.77	12.19	2.56	3.49	1.56	301.20	37,796.83	125.49	194.41	86.94	12.26	51.39	4.19	7.17	3.21
CC043/GeniUnc	Experimental	8	3.81	7.76	2.04	2.79	0.98	208.31	14,197.07	68.15	119.15	42.13	14.93	66.16	4.43	8.13	2.88
CC072/TauUnc	Experimental	8	4.45	19.42	4.37	4.41	1.56	284.94	57,888.53	203.16	240.60	85.07	10.32	55.86	5.41	7.47	2.64

Supporting Table 6. Descriptive statistics for cumulative body mass and composition response over eight weeks of treatment in young CC002/Unc and CC037/TauUnc females.

				Cumulative Body Mass Response					Cumulative Body Fat % Response					Cumulative Lean Mass % Response				
**CC Strain**	**Treatment**	**Timepoint**	**n**	**Mean**	**Variance**	**Coefficient of Variance**	**Std Deviation**	**Std Error**	**Mean**	**Variance**	**Coefficient of Variance**	**Std Deviation**	**Std Error**	**Mean**	**Variance**	**Coefficient of Variance**	**Std Deviation**	**Std Error**
CC002/Unc	Control	1	3	4.02	2.66	0.66	1.63	0.94	9.21	763.23	82.89	27.63	15.95	-1.28	3.87	-3.02	1.97	1.14
CC002/Unc	Control	2	3	3.88	5.02	1.29	2.24	1.29	16.90	1,506.17	89.15	38.81	22.41	-2.64	12.22	-4.64	3.50	2.02
CC002/Unc	Control	3	3	4.66	9.49	2.04	3.08	1.78	23.63	1,215.70	51.44	34.87	20.13	-3.19	10.28	-3.22	3.21	1.85
CC002/Unc	Control	4	3	10.47	52.31	5.00	7.23	4.18	30.92	1,673.27	54.11	40.91	23.62	-4.46	9.63	-2.16	3.10	1.79
CC002/Unc	Experimental	1	9	2.11	13.41	6.34	3.66	1.22	28.94	1,018.77	35.21	31.92	10.64	-2.55	11.63	-4.55	3.41	1.14
CC002/Unc	Experimental	2	9	-0.11	11.02	-104.03	3.32	1.11	20.30	585.70	28.85	24.20	8.07	-2.30	5.23	-2.27	2.29	0.76
CC002/Unc	Experimental	3	9	-0.07	30.55	-467.03	5.53	1.84	25.61	1,221.03	47.68	34.94	11.65	-1.98	20.35	-10.26	4.51	1.50
CC002/Unc	Experimental	4	9	3.97	47.43	11.94	6.89	2.30	31.95	1,067.06	33.39	32.67	10.89	-2.67	12.15	-4.54	3.49	1.16
CC037/TauUnc	Control	1	5	4.09	31.29	7.64	5.59	2.50	8.65	451.28	52.20	21.24	9.50	-0.98	10.34	-10.59	3.22	1.44
CC037/TauUnc	Control	2	5	2.02	87.05	43.02	9.33	4.17	-0.77	903.66	-1,170.73	30.06	13.44	0.40	18.22	45.91	4.27	1.91
CC037/TauUnc	Control	3	5	4.35	91.65	21.05	9.57	4.28	19.04	1,393.71	73.22	37.33	16.70	-2.03	24.58	-12.08	4.96	2.22
CC037/TauUnc	Control	4	5	5.52	74.65	13.53	8.64	3.86	14.77	837.61	56.71	28.94	12.94	-1.57	22.28	-14.17	4.72	2.11
CC037/TauUnc	Experimental	1	7	4.66	39.03	8.38	6.25	2.36	6.68	296.53	44.38	17.22	6.51	-1.62	6.60	-4.08	2.57	0.97
CC037/TauUnc	Experimental	2	7	5.12	29.80	5.83	5.46	2.06	-5.60	136.33	-24.36	11.68	4.41	-0.40	4.59	-11.43	2.14	0.81
CC037/TauUnc	Experimental	3	7	5.55	18.08	3.26	4.25	1.61	17.88	706.26	39.50	26.58	10.04	-2.98	3.96	-1.33	1.99	0.75
CC037/TauUnc	Experimental	4	7	4.50	48.58	10.80	6.97	2.63	0.19	407.91	2,145.86	20.20	7.63	0.14	14.66	106.55	3.83	1.45

Supporting Table 7. Descriptive statistics of physical activity traits in CC002/Unc and CC037/TauUnc young females.

				Total Distance (km)					Total Duration (1-min)					Mean Speed (m/min)				
CC Strain	Treatment	Timepoint	n	Mean	Variance	Coefficient of Variance	Std Deviation	Std Error	Mean	Variance	Coefficient of Variance	Std Deviation	Std Error	Mean	Variance	Coefficient of Variance	Std Deviation	Std Error
CC002/Unc	Experimental	1	9	49.66	415.77	8.37	20.39	6.80	3,485.89	625,308.36	179.38	790.76	263.59	13.38	13.50	1.01	3.67	1.22
CC002/Unc	Experimental	2	9	52.42	770.45	14.70	27.76	9.25	3,487.44	1,041,438.53	298.63	1,020.51	340.17	14.41	16.65	1.16	4.08	1.36
CC002/Unc	Experimental	3	9	32.01	111.79	3.49	10.57	3.52	2,434.67	177,645.50	72.97	421.48	140.49	13.09	14.19	1.08	3.77	1.26
CC002/Unc	Experimental	4	9	29.77	220.83	7.42	14.86	4.95	2,315.56	516,770.03	223.17	718.87	239.62	12.59	14.42	1.15	3.80	1.27
CC037/TauUnc	Experimental	1	7	72.13	752.68	10.44	27.44	10.37	4,297.86	860,606.48	200.24	927.69	350.63	15.50	18.30	1.18	4.28	1.62
CC037/TauUnc	Experimental	2	7	95.22	660.17	6.93	25.69	9.71	4,932.43	871,797.95	176.75	933.70	352.91	18.15	18.54	1.02	4.31	1.63
CC037/TauUnc	Experimental	3	7	87.94	473.71	5.39	21.76	8.23	4,319.86	248,753.14	57.58	498.75	188.51	20.14	13.71	0.68	3.70	1.40
CC037/TauUnc	Experimental	4	7	82.23	349.63	4.25	18.70	7.07	3,998.43	163,387.62	40.86	404.21	152.78	20.10	13.06	0.65	3.61	1.37

Supporting Table 8. Descriptive statistics for body mass and composition response across four CC strains, both sexes and three exercise training programs.

				Body Mass Response					Body Fat % Response					Lean Mass % Response				
CC Strain	Sex	Intensity	n	Mean	Variance	Coefficient of Variance	Std Deviation	Std Error	Mean	Variance	Coefficient of Variance	Std Deviation	Std Error	Mean	Variance	Coefficient of Variance	Std Deviation	Std Error
CC002/Unc	F	HIIT	14	11.68	44.22	3.79	6.65	1.78	-7.53	565.02	-75.07	23.77	6.35	0.34	11.39	33.87	3.37	0.90
CC002/Unc	F	MICT	16	11.28	46.33	4.11	6.81	1.70	15.84	1,146.68	72.39	33.86	8.47	-2.25	20.83	-9.28	4.56	1.14
CC002/Unc	F	NE	8	15.79	7.78	0.49	2.79	0.99	16.85	555.60	32.98	23.57	8.33	-2.27	6.91	-3.05	2.63	0.93
CC002/Unc	M	HIIT	6	6.76	9.45	1.40	3.07	1.26	-15.18	471.42	-31.06	21.71	8.86	-0.70	4.23	-6.08	2.06	0.84
CC002/Unc	M	MICT	8	7.54	10.80	1.43	3.29	1.16	-13.80	696.30	-50.47	26.39	9.33	0.66	1.01	1.53	1.00	0.36
CC002/Unc	M	NE	7	8.64	33.43	3.87	5.78	2.19	-9.85	181.14	-18.39	13.46	5.09	0.08	4.76	62.36	2.18	0.82
CC013/GeniUnc	F	HIIT	4	3.58	34.07	9.52	5.84	2.92	13.34	523.78	39.25	22.89	11.44	-1.16	2.09	-1.79	1.44	0.72
CC013/GeniUnc	F	MICT	8	5.09	11.11	2.18	3.33	1.18	2.54	1,186.18	466.31	34.44	12.18	-0.71	3.65	-5.12	1.91	0.68
CC013/GeniUnc	F	NE	8	12.83	15.18	1.18	3.90	1.38	47.35	3,024.06	63.87	54.99	19.44	-2.52	5.24	-2.08	2.29	0.81
CC013/GeniUnc	M	HIIT	8	4.11	11.14	2.71	3.34	1.18	20.78	1,948.33	93.77	44.14	15.61	-1.53	3.30	-2.16	1.82	0.64
CC013/GeniUnc	M	MICT	8	8.41	49.16	5.84	7.01	2.48	9.59	2,065.23	215.24	45.44	16.07	-1.26	6.71	-5.32	2.59	0.92
CC013/GeniUnc	M	NE	8	15.83	17.08	1.08	4.13	1.46	37.52	2,054.29	54.76	45.32	16.02	-2.42	4.29	-1.78	2.07	0.73
CC027/GeniUnc	F	HIIT	14	6.12	18.34	3.00	4.28	1.14	-1.69	1,782.88	-1,056.28	42.22	11.28	-1.45	3.41	-2.36	1.85	0.49
CC027/GeniUnc	F	MICT	15	7.18	13.38	1.86	3.66	0.94	29.77	3,627.30	121.86	60.23	15.55	-2.54	4.74	-1.87	2.18	0.56
CC027/GeniUnc	F	NE	8	15.69	24.30	1.55	4.93	1.74	-8.24	2,022.77	-245.37	44.98	15.90	0.25	2.47	9.79	1.57	0.56
CC027/GeniUnc	M	HIIT	11	3.70	14.95	4.04	3.87	1.17	37.36	7,058.99	188.93	84.02	25.33	-2.40	13.20	-5.51	3.63	1.10
CC027/GeniUnc	M	MICT	11	6.20	21.81	3.52	4.67	1.41	12.63	1,989.01	157.45	44.60	13.45	-1.72	10.18	-5.91	3.19	0.96
CC027/GeniUnc	M	NE	8	14.61	18.06	1.24	4.25	1.50	38.68	5,447.14	140.83	73.80	26.09	-0.78	2.66	-3.43	1.63	0.58
CC037/TauUnc	F	HIIT	15	3.74	38.43	10.27	6.20	1.60	13.68	2,118.10	154.83	46.02	11.88	-2.12	12.55	-5.92	3.54	0.91
CC037/TauUnc	F	MICT	15	8.17	38.67	4.73	6.22	1.61	78.98	3,900.85	49.39	62.46	16.13	-8.46	23.65	-2.80	4.86	1.26
CC037/TauUnc	F	NE	7	10.29	23.78	2.31	4.88	1.84	38.34	1,583.78	41.31	39.80	15.04	-3.49	12.90	-3.69	3.59	1.36
CC037/TauUnc	M	HIIT	11	8.37	42.83	5.12	6.54	1.97	26.14	1,410.58	53.96	37.56	11.32	-3.36	24.44	-7.27	4.94	1.49
CC037/TauUnc	M	MICT	11	9.30	19.86	2.14	4.46	1.34	36.31	1,576.17	43.41	39.70	11.97	-3.57	19.01	-5.32	4.36	1.31
CC037/TauUnc	M	NE	8	9.54	29.66	3.11	5.45	1.93	15.26	267.58	17.54	16.36	5.78	-1.89	1.63	-0.86	1.28	0.45
