# Supplementary material for: CC002/Unc females are mouse models of exercise‐induced paradoxical fat response
Source: Physiol Rep. 2018 Jun 19;6(12):e13716. doi: 10.14814/phy2.13716 (PMC6009762; doi:10.14814/phy2.13716)

Supporting Figure 1

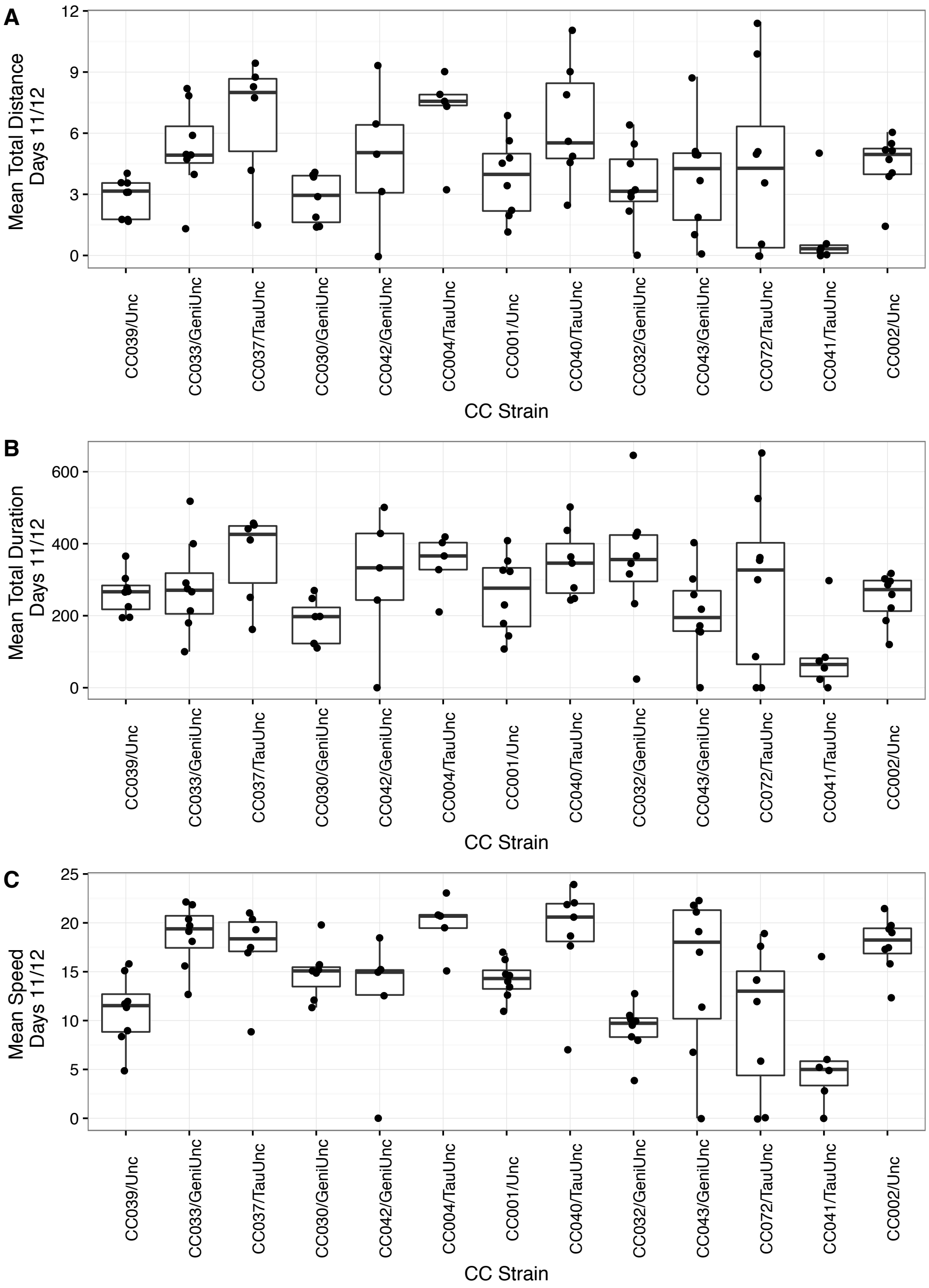

Supporting Figure 2

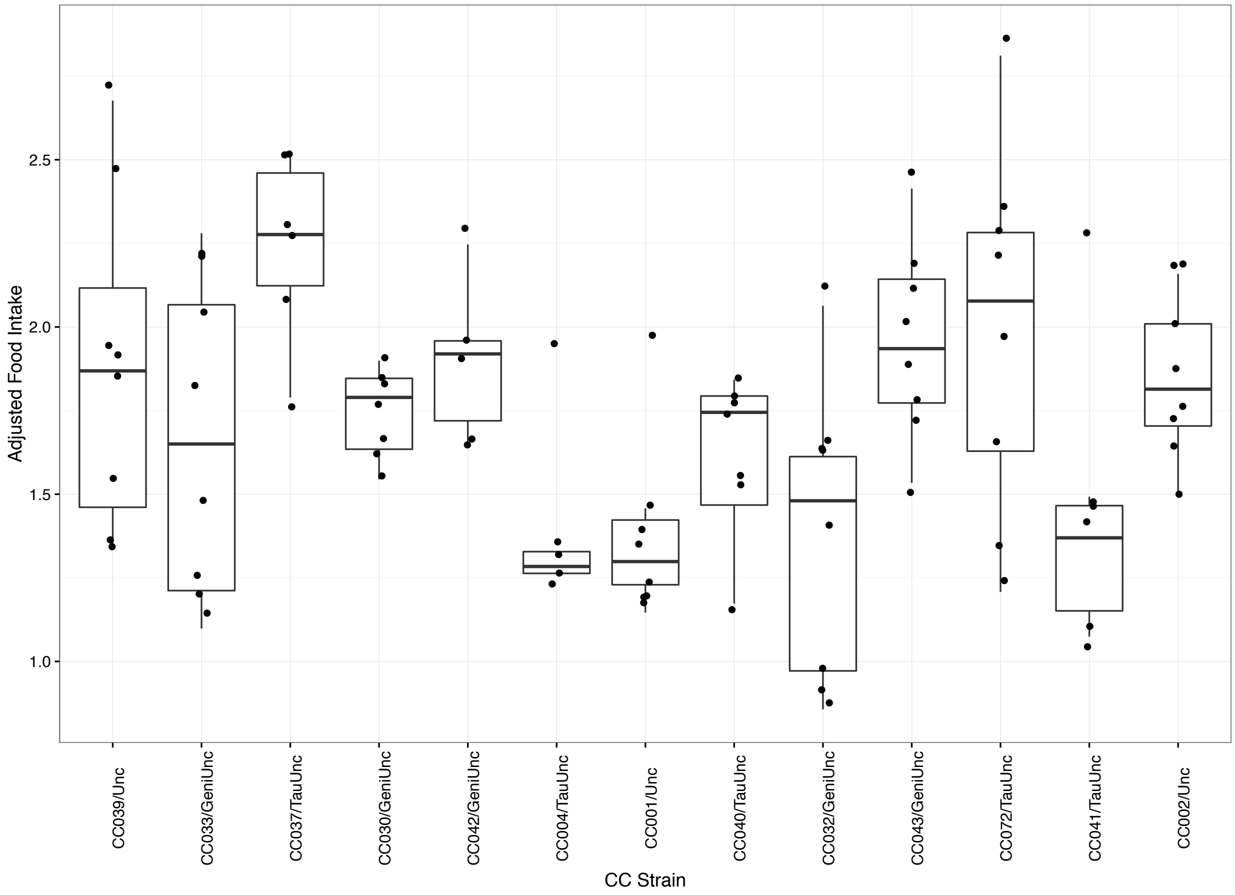

Supporting Figure 3:

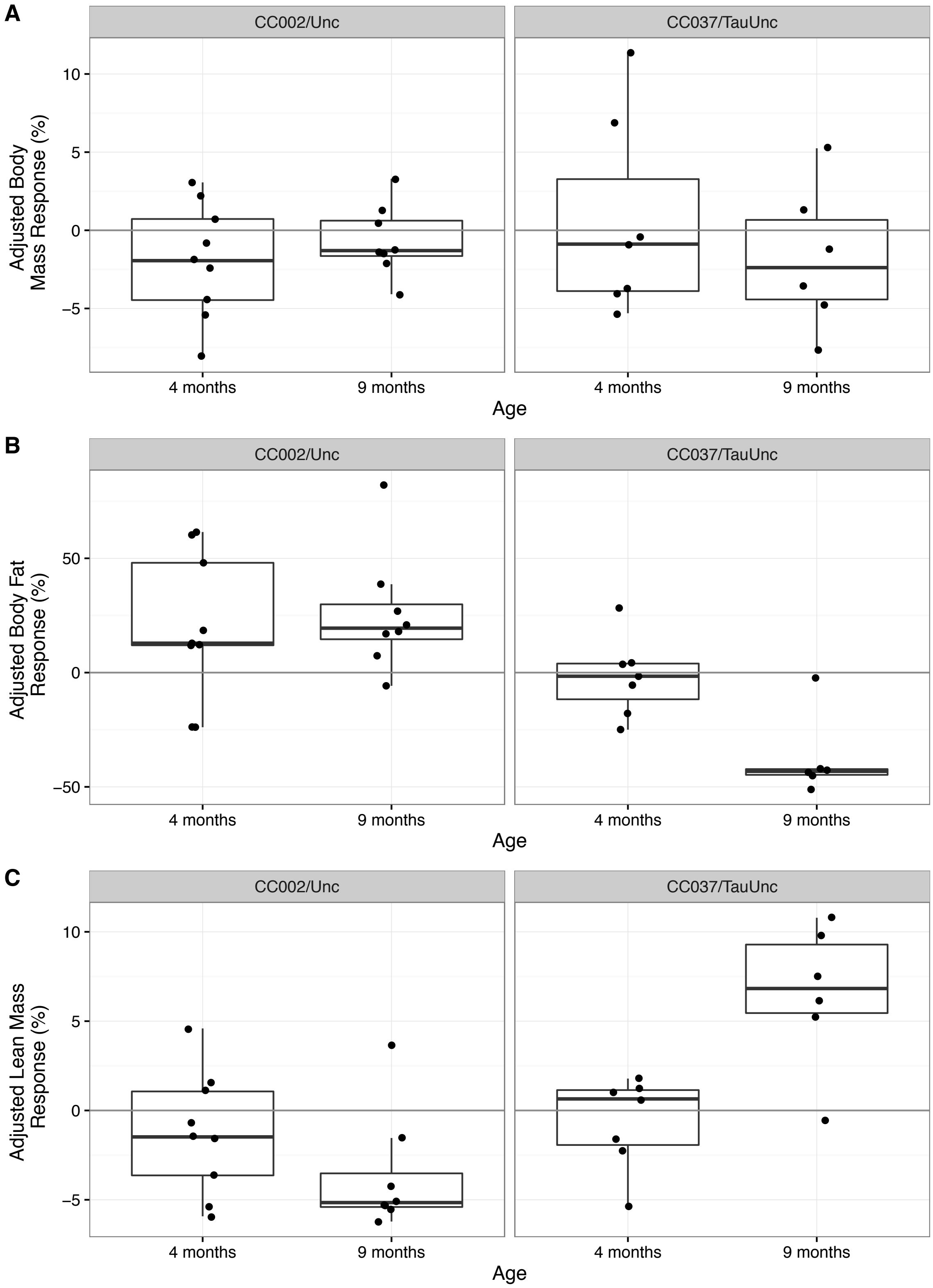

Supporting Figure 4

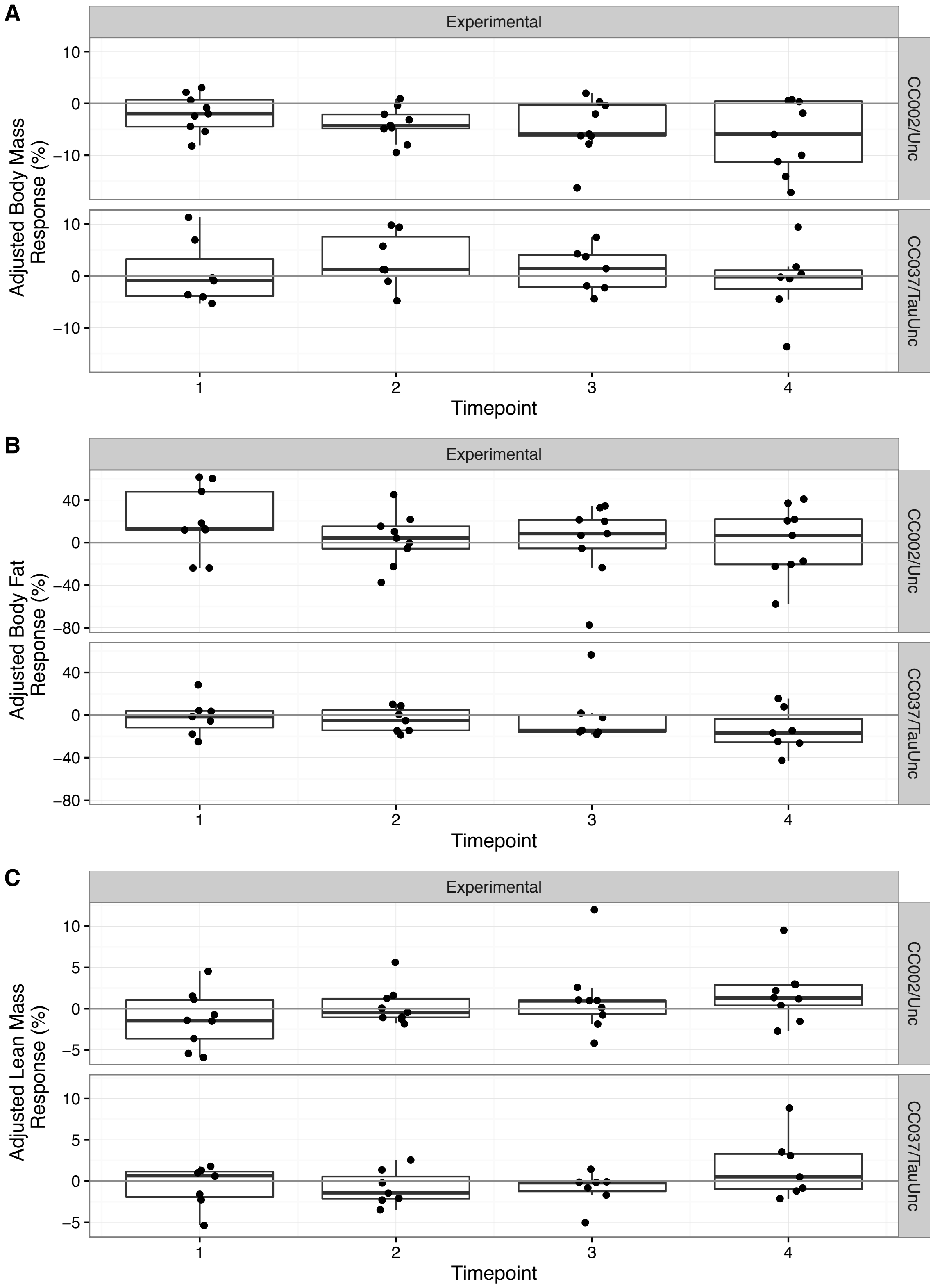

Supporting Figure 5

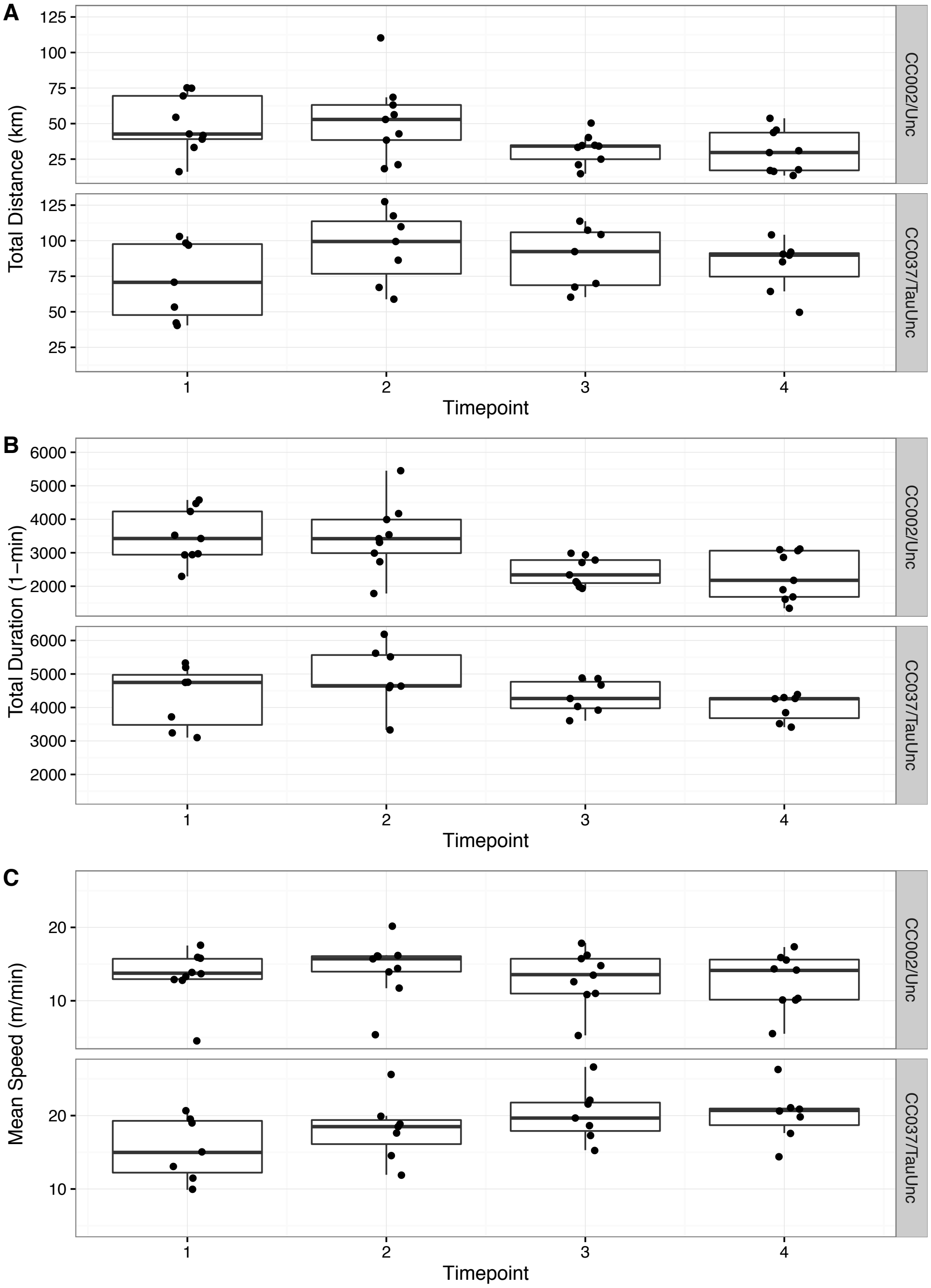

Supporting Figure 6

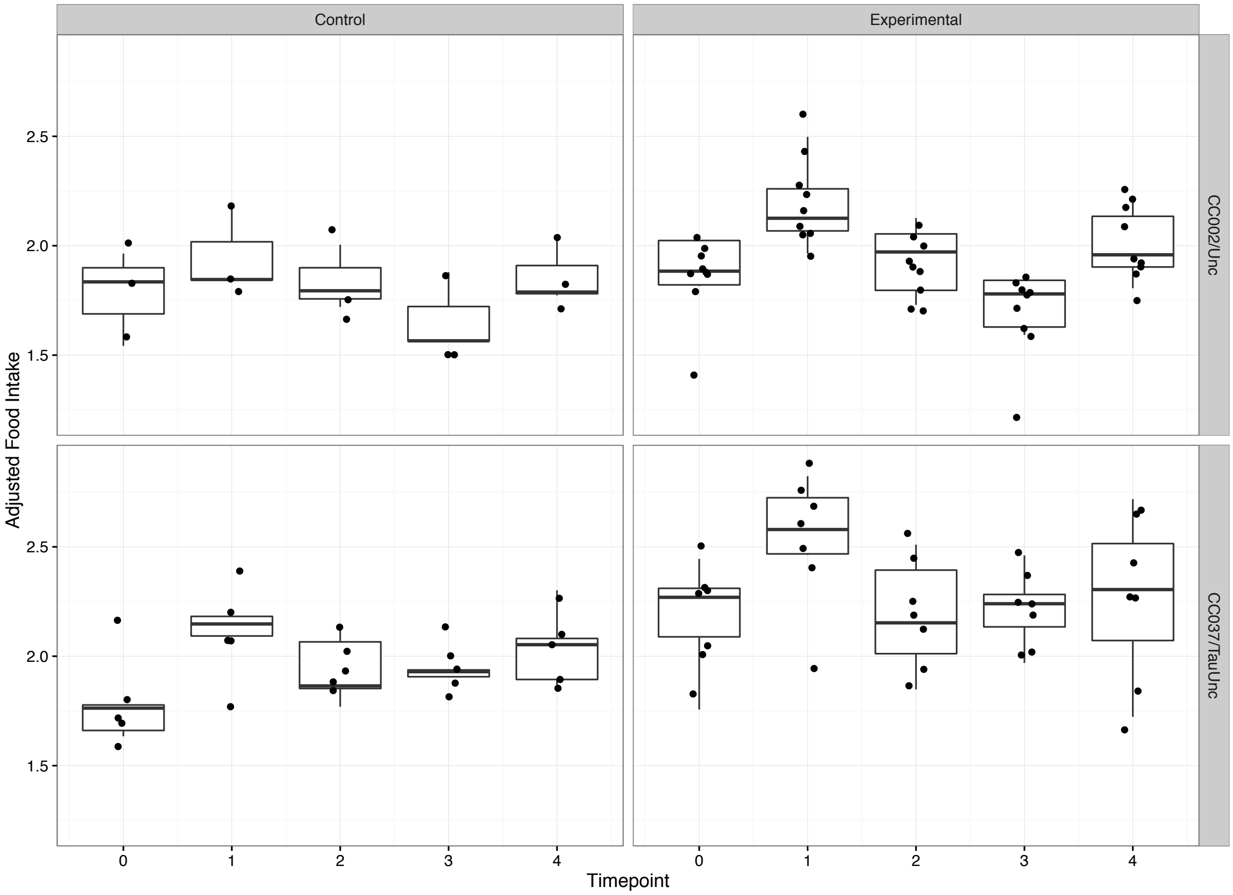

Supplement: Supplementary file 2 — Figure S1. Physical activity traits in aged females across 13 CC strains. Figure S2. Adjusted food intake in experimental cohort across 13 CC strains. Figure S3. Adjusted body mass and composition response to 2 weeks of exercise in young and old CC002/Unc and CC037/TauUnc female mice. Figure S4. Cumulative adjusted exercise‐induced body mass and composition response over 8 weeks in CC002/Unc and CC037/TauUnc female mice. Figure S5. Physical activity traits over 8 weeks of wheel access in CC002/Unc and CC037/TauUnc female mice. Figure S6. Adjusted food intake over 10 weeks of treatment in CC002/Unc and CC037/TauUnc female mice. [file PHY2-6-e13716-s002.pdf]
