# Supplementary material for: CC002/Unc females are mouse models of exercise‐induced paradoxical fat response
Source: Physiol Rep. 2018 Jun 19;6(12):e13716. doi: 10.14814/phy2.13716 (PMC6009762; doi:10.14814/phy2.13716)
Supplement: Supplementary file 3 [file PHY2-6-e13716-s003.docx]

Supporting Figure legends:

Supporting Figure 1: Physical activity traits in aged females across 13 CC strains. Mean total distance (km) (A), mean total duration (1-min intervals) (B) and mean speed (m/min) (C) for days 11/12 of wheel access. Each dot represents and individual female mouse. Strains are ordered by median adjusted fat response.

Supporting Figure 2: Adjusted food intake in experimental cohort across 13 CC strains. Adjusted food intake to two weeks of treatment in mice from the experimental cohort. Each dot represents an individual female mouse. Strains are ordered by median adjusted fat response.

Supporting Figure 3: Adjusted body mass and composition response to two weeks of exercise in young and old CC002/Unc and CC037/TauUnc female mice. Adjusted body mass response (%) (A), adjusted body fat percentage response (%) (B) and adjusted lean mass percentage response (%) (C) to two weeks of wheel access in the experimental cohorts. Responses are adjusted to mean responses in the control cohort. Each dot represents an individual female mouse.

Supporting Figure 4: Cumulative adjusted exercise-induced body mass and composition response over 8 weeks in CC002/Unc and CC037/TauUnc female mice. Adjusted body mass response (%) (A), adjusted body fat percentage response (%) (B) and adjusted lean mass percentage response (%) (C) for each two week interval of the experiment in the experimental treatment cohort. Responses are adjusted to mean responses in the control cohort. Each dot represents an individual female mouse. Each response is calculated for a two week interval. Responses are represented as timepoint intervals: timepoint 0 (single housing acclimation), 1 (weeks 0-2 of treatment), 2 (weeks 2-4 of treatment), 3 (weeks 4-6 of treatment) and 4 (weeks 6-8 of treatment).

Supporting Figure 5: Physical activity traits over 8 weeks of wheel access in CC002/Unc and CC037/TauUnc female mice. Total distance (km) (A), total duration (1-min intervals) (B) and mean speed (m/min) (C) are represented in two week intervals over 8 weeks of wheel access. Each dot represents and individual female mouse. Timepoint intervals: timepoint 1 (weeks 0-2 of wheel access), 2 (weeks 2-4 of wheel access), 3 (weeks 4-6 of wheel access) and 4 (weeks 6-8 of wheel access).

Supporting Figure 6: Adjusted food intake over 10 weeks of treatment in CC002/Unc and CC037/TauUnc female mice. Adjusted food intake for each two week intervals of treatment (control or experimental). Each dot represents an individual female mouse. Timepoint intervals are timepoint 0 (single housing acclimation), 1 (weeks 0-2 of treatment), 2 (weeks 2-4 of treatment), 3 (weeks 4-6 of treatment) and 4 (weeks 6-8 of treatment).
